# Synthesis and NMR studies of malonyl-linked glycoconjugates of *N*-(2-aminoethyl)glycine. Building blocks for the construction of combinatorial glycopeptide libraries

**DOI:** 10.3762/bjoc.12.183

**Published:** 2016-08-30

**Authors:** Markus Nörrlinger, Sven Hafner, Thomas Ziegler

**Affiliations:** 1 Institute of Organic Chemistry, University of Tuebingen, Auf der Morgenstelle 18, 72076 Tuebingen, Germany

**Keywords:** amino acids, carbohydrates, glycoconjugates, glycopeptides, *N*-(2-aminoethyl)glycine

## Abstract

Four glycoconjugate building blocks for the construction of combinatorial PNA like glycopeptide libraries were prepared in 75–79% yield by condensing *tert*-butyl *N*-[2-(*N*-9-fluorenylmethoxycarbonylamino)ethyl]glycinate (AEG) **5** with 3-oxo-3-(2,3,4,6-tetra-*O*-acetyl-β-D-glucopyranosylamino)- (**6a**), 3-oxo-3-(β-D-galactopyranosylamino)- (**6b**), 3-oxo-3-(2-acetamido-2-deoxy-3,4,6-tetra-*O*-acetyl-β-D-glucopyranosylamino)- (**6c**) and 3-oxo-3-(2-acetamido-2-deoxy-3,4,6-tetra-*O*-acetyl-β-D-galactopyranosylamino)propanoic acid (**6d)**, respectively. The resulting AEG glycoconjugates **1a–d** were converted into the corresponding free acids **2a–d** in 97–98% yield by treatment with aqueous formic acid. The Fmoc group of compound **1c** was removed and the intermediate amine **9** was condensed with **2a** to afford the corresponding glycosylated AEG dipeptide **4** in 58% yield. All glycoconjugate building blocks showed the presence of *cis* and *trans* rotamers. Compounds **1a**, **1b** and **4** were subjected to temperature dependent ^1^H NMR spectroscopy in order to determine the coalescence temperature which resulted in calculated rotation barriers of 17.9–18.3 kcal/mol for the rotamers.

## Introduction

The glycocalyx is a fringy or fuzzy polysaccharide layer coating most animal and many bacterial cells. It is covalently bound to the surface of the cell membrane through glycoproteins and plays a major role in numerous biologically important recognition mechanisms like cell–cell recognition, signal transduction and immunological processes [1–4]. Therefore, investigating the delicate carbohydrate–protein interactions on a molecular level is an inalienable prerequisite for a deep understanding of the fundamental cellular recognition processes involving the complex saccharides of the glycocalyx [5–8]. Unfortunately, isolation of pure carbohydrate material or specific glycoconjugates from natural sources remains a difficult, sometimes even an unrealizable task, for naturally occurring saccharides exhibit micro-heterogenity which makes it nearly impossible to obtain pure material from such sources. Chemical or chemoenzymatic syntheses of complex oligosaccharides, on the other hand, may provide sufficient amounts of pure material for such studies. Despite the great achievements in oligosaccharide synthesis during the past decades, the preparation of complex oligosaccharides can be tedious, lengthy or circuitous, and the often intrinsic intricacy of a chemical saccharide synthesis makes it sometimes impossible to prepare a certain saccharide or glycoconjugate [9]. Therefore, gaining access to new glycoconjugates which are easily accessible by chemical synthesis and which are able to mimic the interaction between a specific protein and its natural oligosaccharide ligand are highly desirable [10–13]. In our previous work we introduced various trifunctional glycopeptide building blocks derived from aspartic acid, 3-aminomethyl-5-aminobenzoic acid [14] and from the PNA-like *N*-(2-aminoethyl)glycine (AEG) backbone to which sugar moieties were linked through either simple alkyl chains [15–16], amino alcohols [17–18] or 1,2,3-triazoles [19–21]. These building blocks were used for automated SPOT synthesis on a cellulose surface in order to construct complex glycoconjugates which are able to specifically bind to lectins [15,18,20].

**Figure 1:**
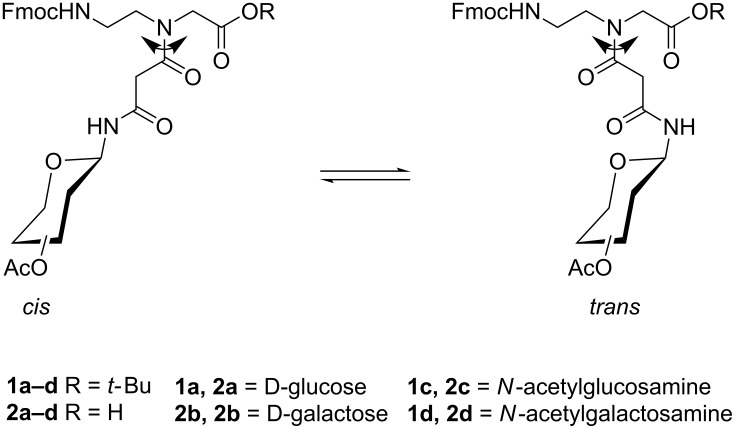
*cis*- and *trans* rotamer of protected PNA building blocks **1a–d** and **2a–d**.

**Figure 2:**
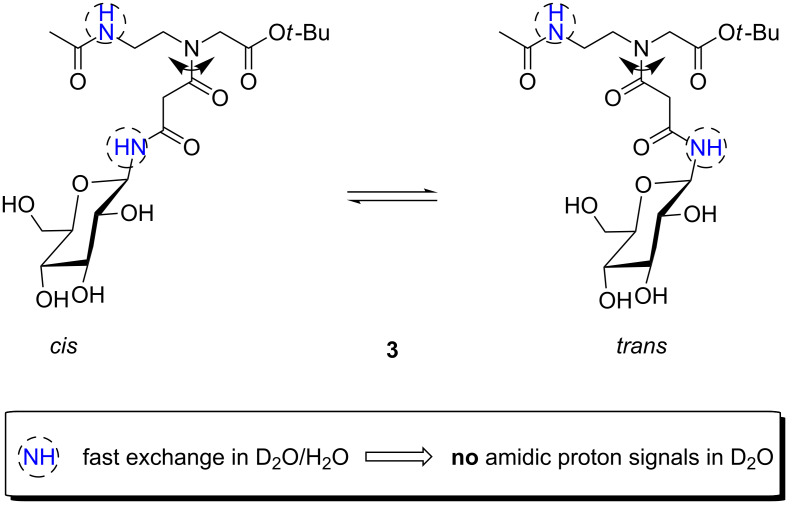
*cis*- and *trans* rotamer of unprotected PNA-building block **3**.

**Figure 3:**
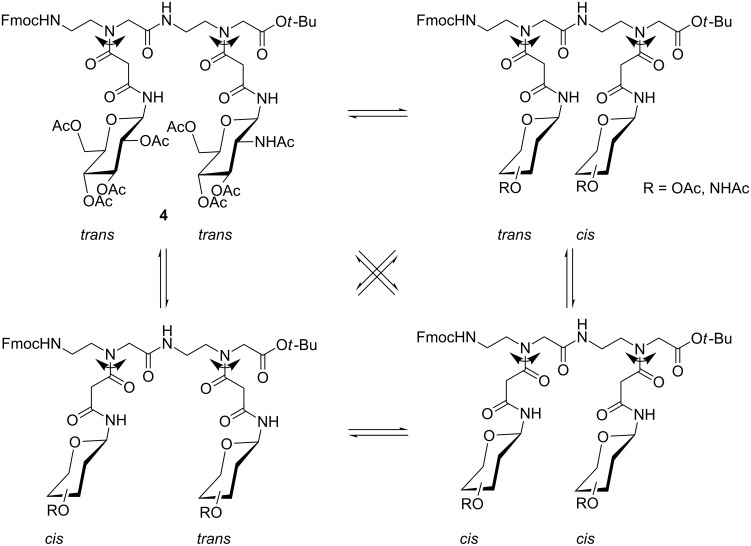
Rotameric structures of dimeric PNA glycoconjugate **4**.

In continuation of these studies, we now describe the preparation of PNA-based glycoconjugate building blocks **1–3** as well as a dimeric glycoconjugate **4** in which the sugar moieties are attached through a malonyl linker (Figures 1–3). For these compounds we studied the *cis*/*trans*-rotameric structures via temperature-dependent ^1^H NMR spectroscopy. Unfortunately, the amidic protons of the rotamers of the unprotected glycoconjugates could not be observed in the ^1^H NMR spectra in D_2_O due to the fast H/D exchange with the solvent (Figure 2). Nevertheless, we could identify two rotameric forms exhibiting a *cis*/*trans* ratio of 2:1 which was in accordance with similar rotamers described in literature [22]. Hereupon, we report on our investigations concerning the structures of the fully protected conjugates **1** and **4** for the rotamers of which around the C–N bond we calculated the respective ΔG^‡^_r_-values.

## Results and Discussion

### Synthesis of building blocks

**Scheme 1:**
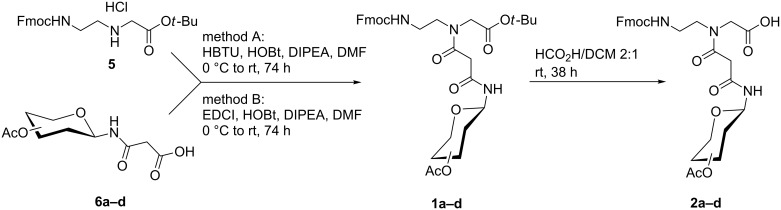
Synthesis of building blocks **1a–d** and **2a–d** (**a** stands for D-glucose, **b** stands for D-galactose, **c** stands for *N*-acetylglucosamine, **d** stands for *N*-acetylgalactosamine).

The preparation of building blocks **1a–d** and **2a–d** started from *tert*-butyl *N*-[2-(*N*-9-fluorenylmethoxycarbonyl)aminoethyl)]glycinate hydrochloride (**5**) which was synthesized in 39% yield from *tert*-butyl bromoacetate according to the procedure published by Thomson et al. [23]. Acetyl protected glycosylaminomalonic acids **6a–d** were prepared from the corresponding *tert*-butyl esters as previously described [14]. Coupling of **5** with acids **6a–d** was achieved with either *N,N,N′,N′*-tetramethyl-*O-*(1*H*-benzotriazol-1-yl)uronium hexafluorophosphate (HBTU) or 1-ethyl-3-(3-dimethylaminopropyl)carbodiimide (EDCI) (Scheme 1, Table 1). In general, HBTU gave higher yields of malonamides **1a–d** than EDCI. Previously, we used EDCI for coupling acids **6a–d** to aniline derivatives because HBTU resulted in byproducts which were difficult to be removed [14]. Such byproducts were not observed here though.

**Table 1:** Synthesis of building blocks **1a–d**.

Entry	Starting material	Coupling method	Product	Yield (%)

1	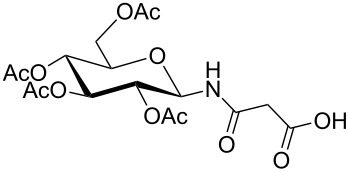 **6a**	A) HBTU B) EDCI	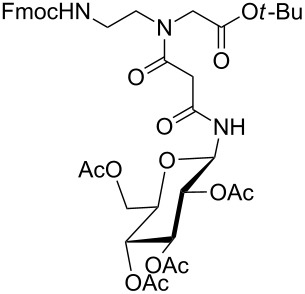 **1a**	A) 79% B) 40%
2	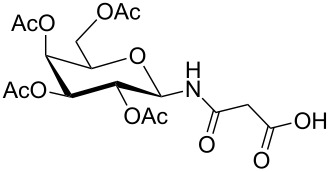 **6b**	A) HBTU B) EDCI	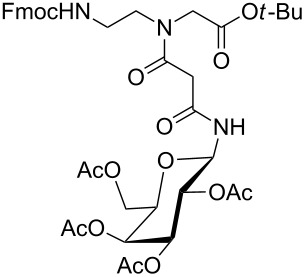 **1b**	A) 79% B) 47%
3	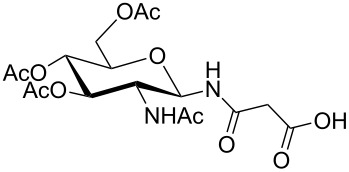 **6c**	A) HBTU B) EDCI	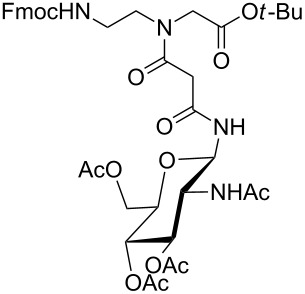 **1c**	A) 75% B) 57%
4	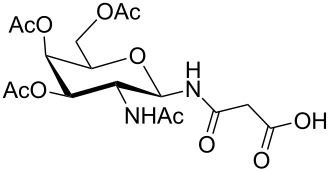 **6d**	A) HBTU B) EDCI	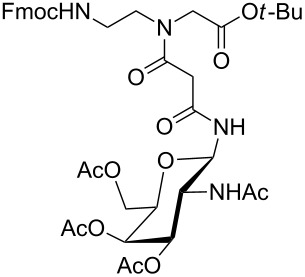 **1d**	A) 77% B) 42%

Next, the *tert*-butyl ester groups of building blocks **1a–d** were removed under acid conditions with a 2:1 mixture of formic acid and dichloromethane at room temperature to give the corresponding free acids **2a–d** in 97–98% yield (Scheme 1, Table 2).

**Table 2:** Synthesis of building blocks **2a–d**.

Entry	Starting material	Product	Yield (%)

1	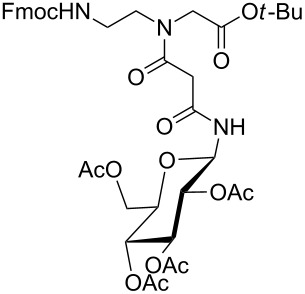 **1a**	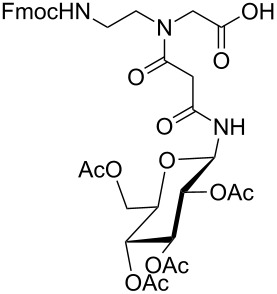 **2a**	98%
2	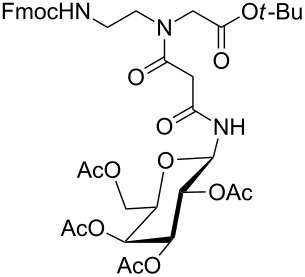 **1b**	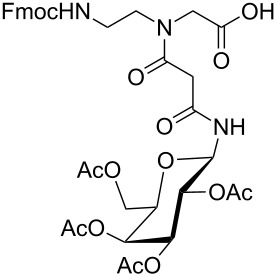 **2b**	97%
3	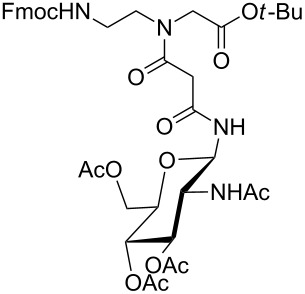 **1c**	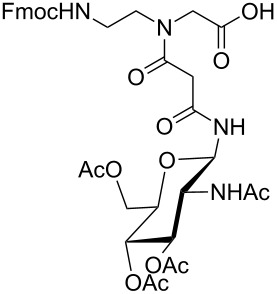 **2c**	97%
4	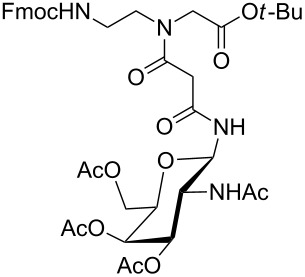 **1d**	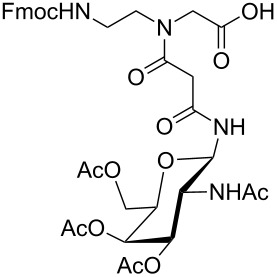 **2d**	97%

**Scheme 2:**
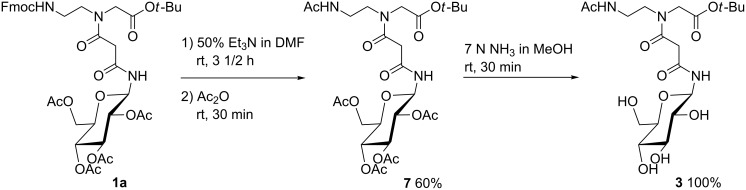
Synthesis of building block **3**.

Partially deprotected building block **3** was prepared from **1a** as follows. First, removal of the Fmoc group in **1a** under basic conditions with triethylamine in DMF gave the crude amino derivate which was acetylated with Ac_2_O to give building block **7** in 60% yield. Removal of the acetyl groups of the sugar moiety in **7** to afford compound **3** could be achieved in a virtually quantitative yield by subjection **7** to a saturated solution of NH_3_ in MeOH (7 N) (Scheme 2).

**Scheme 3:**
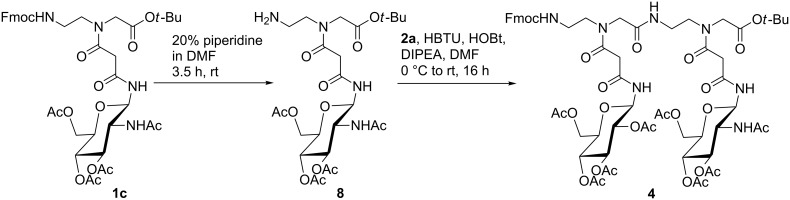
Synthesis of the dimeric glycoconjugate **4**.

In addition to the monomeric glycoconjugates **1a–d** and **2a–d** we also prepared dimer **4** from the glucose containing conjugate **2a** and the *N*-acetylglucosamine containing conjugate **1c**. Treatment of **1c** with 20% piperidine in DMF at room temperature for 3.5 h gave partially protected compound **8** which was used for the next step without further purification. Coupling of **2a** with crude **8** using HBTU, 1-hydroxytriazole (HOBt) and diisopropylethylamine (DIPEA) in DMF gave dipeptide **4** in 58% yield (Scheme 3).

### 1D-NMR investigation

**Figure 4:**
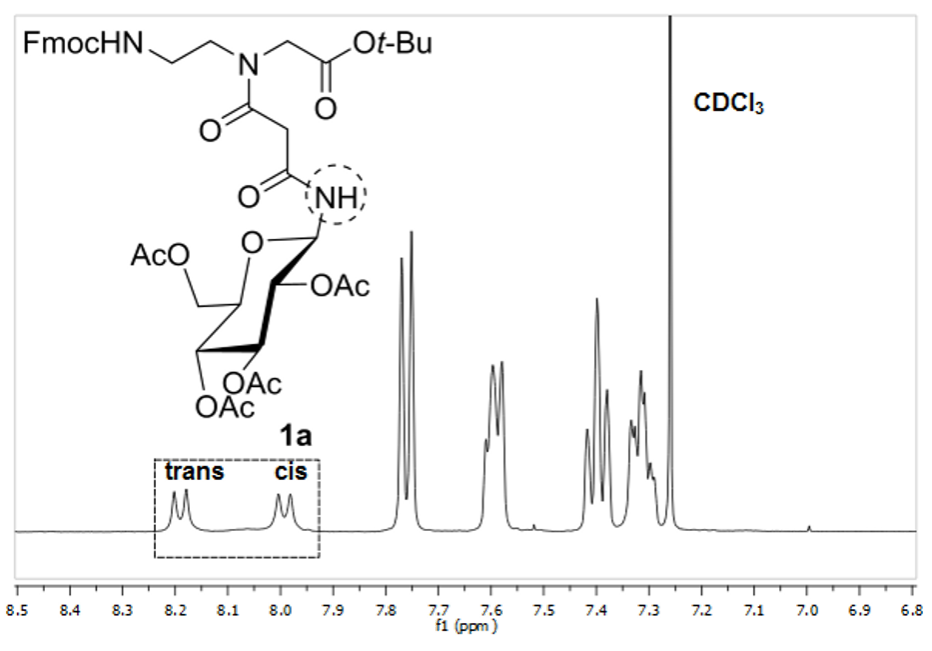
^1^H NMR (400 MHz) of building block **1a** in CDCl_3_. The amide signals of the rotamers are marked.

**Figure 5:**
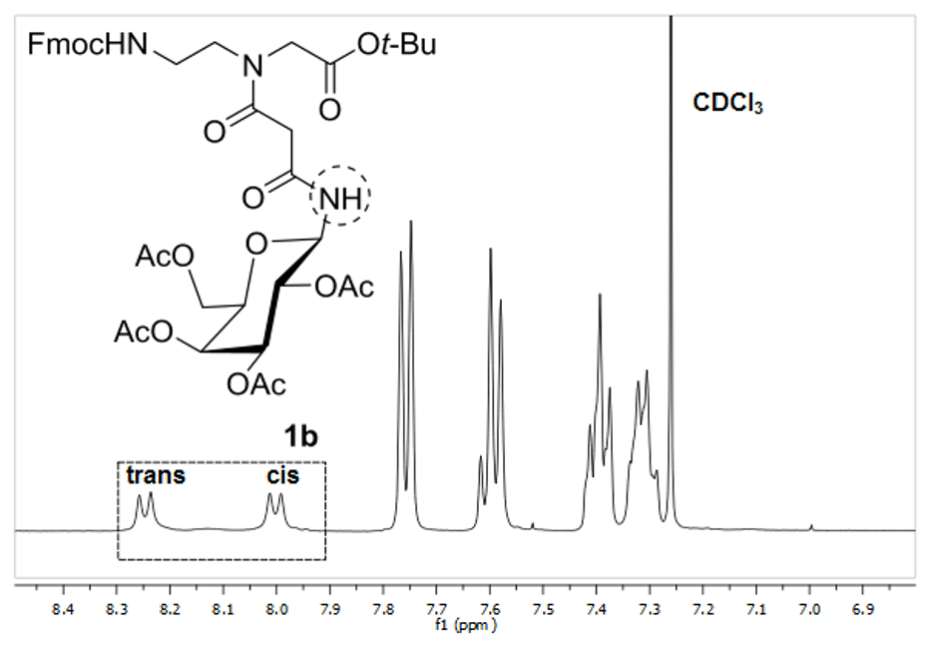
^1^H NMR (400 MHz) of building block **1b** in CDCl_3_. The amide signals of the rotamers are marked.

Glycoconjugates **1a**,**b** and **4** were submitted to temperature-dependent ^1^H NMR spectroscopy in order to reveal, verify and determine their *cis*- and *trans*-rotameric structures (see also Figures 1–3). The ^1^H NMR spectra of **1a** and **1b** in CDCl_3_ at room temperature revealed two separate doublets at 8.19 and 7.99 ppm for **1a** and 8.26 and 7.99 ppm for **1b**, respectively for the anomeric amide proton indicating the presence of two rotamers (Figure 4 and Figure 5). The signals at lower field were assigned to the respective *trans* rotamers whereas the signals at higher field were assigned to the corresponding *cis* rotamers of **1a** and **1b** (see 2D NMR investigations below).

**Figure 6:**
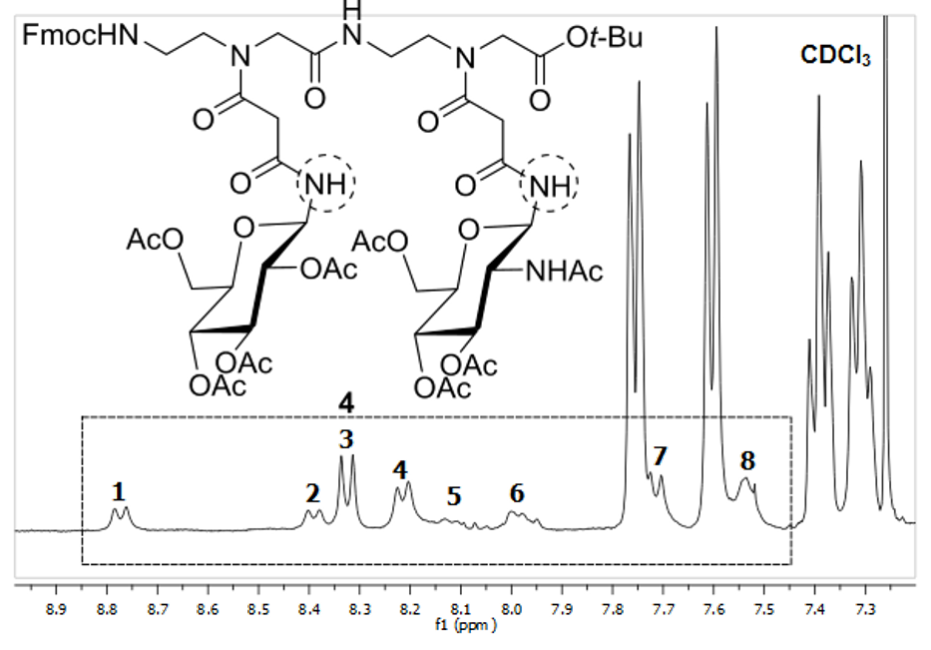
^1^H NMR (400 MHz) of PNA glycoconjugate **4** in CDCl_3_. The amide signals of the rotamers of **4** (2 doublets for each rotamer) are marked 1/2, 3/4, 5/6 and 7/8.

The ^1^H NMR spectrum of the dimeric PNA glycoconjugate **4** in CDCl_3_ showed the presence of four different rotameric structures (see also Figure 3). This was evident from eight distinct doublet signals for the anomeric amide protons (Figure 6). Here, no unambiguous assignment of the observed doublets to respective *cis*/*trans* rotameric forms could be achieved by 2D NMR spectroscopy.

**Figure 7:**
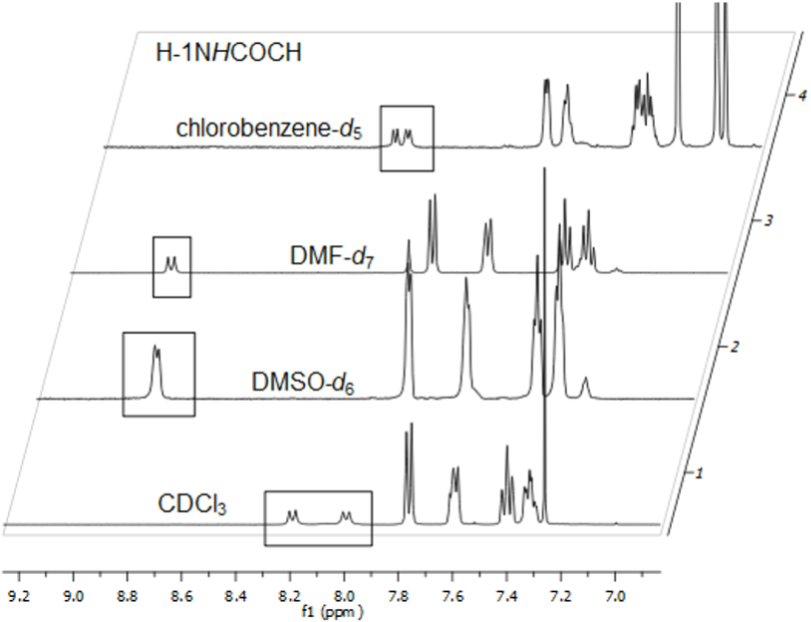
Building block **1a** in CDCl_3_, DMSO-*d*_6_, DMF-*d*_7_ and chlorobenzene-*d*_5_*.*

**Figure 8:**
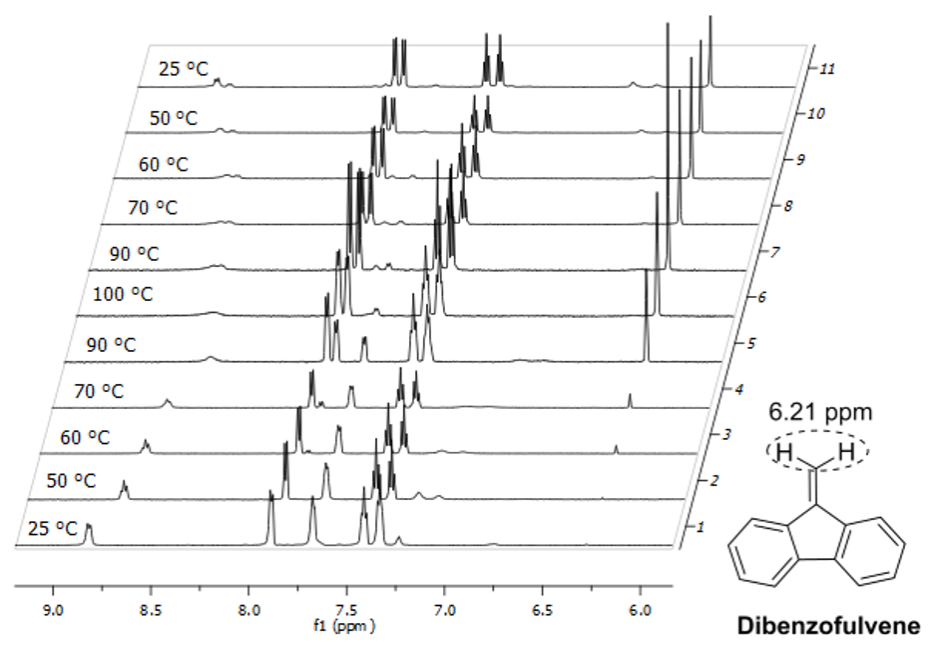
Temperature-dependent ^1^H NMR (600 MHz) spectra of building block **1a** in DMSO-*d*_6_*.*

Since deuterochloroform is not suitable for temperature-dependent ^1^H NMR experiments at higher temperatures which, in turn, are necessary for determining the coalescence temperature of both rotamers we measured the ^1^H NMR of **1a** in DMSO-*d*_6_, DMF-*d*_7_, and chlorobenzene-*d*_5_ as well (Figure 7). In DMSO-*d*_6_ and DMF-*d*_7_ which both are common solvents for temperature-dependent NMR spectroscopy [24] the anomeric amide protons of the two rotamers of **1a** were not sufficiently separated. Furthermore, Fmoc groups are known to be unstable in DMSO and DMF at higher temperatures [24–25]. Indeed, when **1a** was heated in DMSO-*d*_6_ above 60 °C a signal of dibenzofulvene at 6.21 ppm appeared, indicating the cleavage of the Fmoc group in **1a** (Figure 8). In chlorobenzene-*d*_5_ (bp 131 °C), however, sufficient separation of the two anomeric amide protons of the rotamers of **1a** were observed and no cleavage of the Fmoc group occurred. Höck et al. [24] could also show that chlorobenze-*d*_5_ does not cause cleavage of Fmoc groups in peptides up to 120 °C.

**Figure 9:**
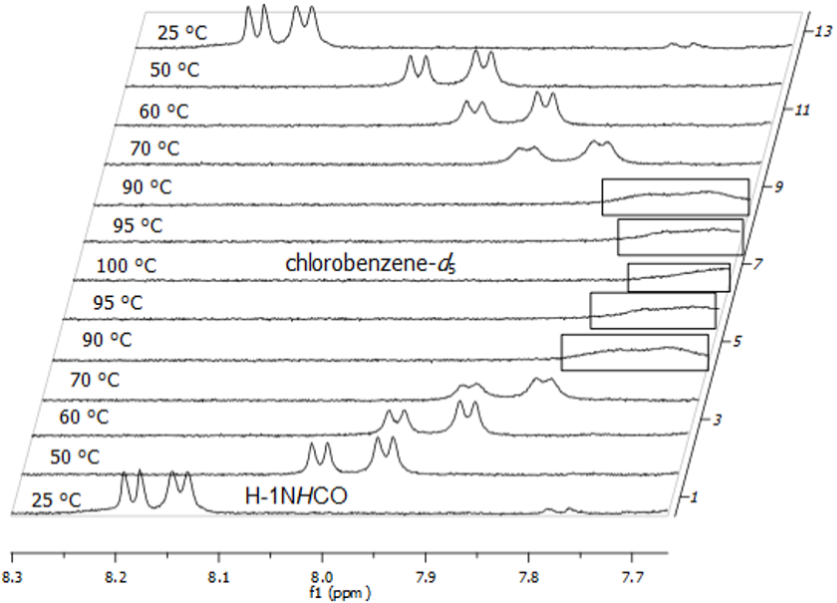
Temperature-dependent ^1^H NMR (600 MHz) spectra of building block **1a** in chlorobenzene-*d*_5_, broadened signals near the coalescence temperature are marked.

Figure 9 shows the temperature-dependent ^1^H NMR spectra (7.65–8.30 ppm area only) of **1a** in the temperature range between 25 and 100 °C. Heating the sample caused a downfield shift of the anomeric amide protons with coalescence (*T*_c_) at 90–95 °C (363–368 K). Unfortunately, the amide signals partially overlapped with the proton signals of the Fmoc group at the coalescence temperature so that no exact coalescence temperature could be determined.

**Figure 10:**
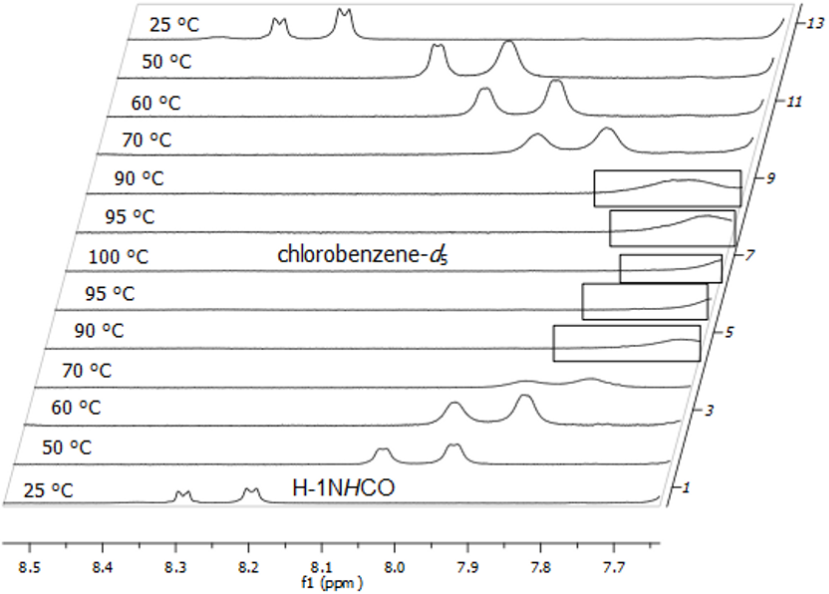
Temperature-dependent ^1^H NMR (600 MHz) spectra of building block **1b** in chlorobenzene-*d*_5_, broadened signals near the coalescence temperature are marked.

Figure 10 shows the temperature-dependent ^1^H NMR spectra (7.65–8.50 ppm area only) of **1b** in the range between 25 and 100 °C. In this case the corresponding coalescence temperature (*T*_c_) could be determined at 90–95 °C (363–368 K).

**Figure 11:**
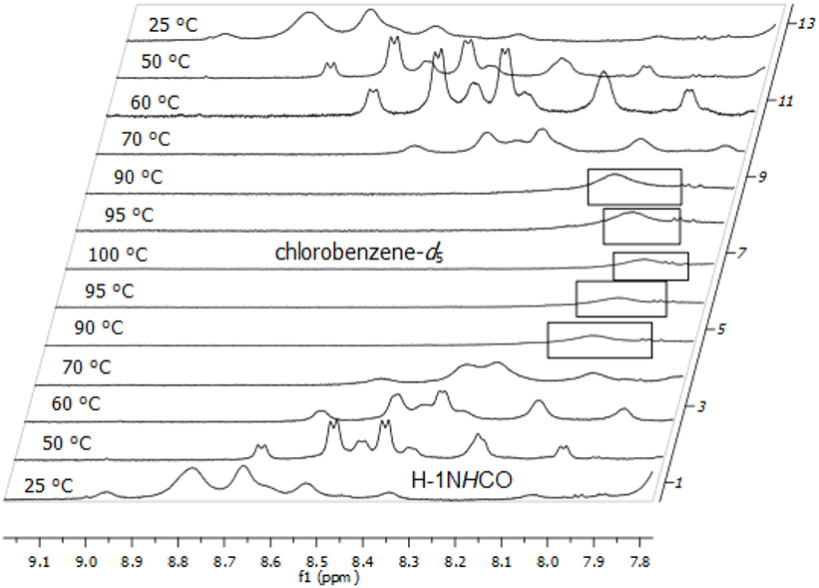
Temperature-dependent ^1^H NMR (600 MHz) spectra of dimeric glycoconjugate **4** in chlorobenzene-*d*_5_, broadened signals near the coalescence temperature are marked.

Figure 11 finally shows the temperature-dependent ^1^H NMR spectra (9.10–7.80 ppm are only) of **4** in the range between 25 and 100 °C. In this case, the corresponding coalescence temperature (*T*_c_) could also be determined at 90–95 °C (363–368 K).

### 2D-NMR investigation

**Figure 12:**
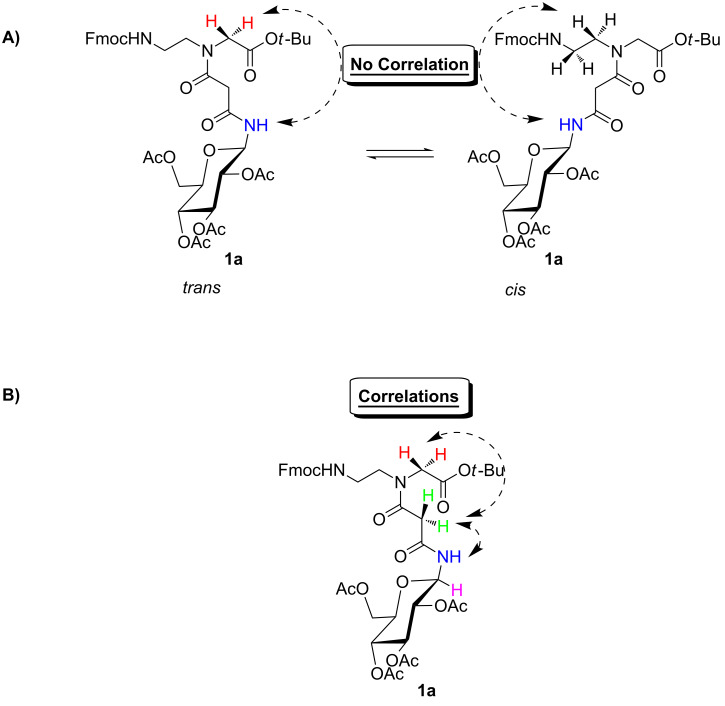
A) Missing correlation between the amidic proton and the methylene or 2-aminoethyl protons of the PNA backbone; B) Observed NOE-correlations of PNA building block **1a**.

In order to unambiguously assign the anomeric amide signals (doublets) from the proton NMR spectra above to the corresponding *cis*/*trans* rotamers of **1a** we measured the NOESY spectrum of conjugate **1a** in CDCl_3_ (see Supporting Information File 1). The spectrum indicates that the direct assignment of the amidic doublets to the respective *cis*- or *trans* rotamers is not possible due to missing crosspeaks between the amidic signals and the methylene or 2-aminoethyl protons of the PNA backbone (Figure 12).

Nevertheless, crosspeaks between the amidic protons and the malonyl protons could be observed, and were used for an indirect assignment of the amidic doublets. Stronger NOE crosspeaks between the methylene protons of the side chain (3.20 ppm) and the methylene protons of the PNA backbone (3.93 ppm) indicated that the distance between these protons should be shorter in comparison to the distance of the protons with a chemical shift at 3.36 and 3.93 ppm. Therefore, the signal of the protons at 3.20 ppm and the doublet at 8.18 ppm belong to the *trans* rotamer whereas the doublet at 8.01 ppm should belong to the *cis* rotameric structure.

### Calculation of Δ*G*^‡^_r_-values

In order to evaluate the rotation barrier around the tertiary peptide bond (C–N bond) we calculated the corresponding Δ*G*^‡^_r_ values for building blocks **1a**,**b** from the measured coalescence temperatures (*T*_c_) by using the Eyring model [26]. The Δ*G*^‡^_c_ values of the dimeric glycoconjugate **4** could not be determined though because no unambiguous assignment of the rotameric structures to the corresponding specific proton signals could be made. Nevertheless, the rotation barriers of glycoconjugate **4** should be rather similar to the barriers of building blocks **1a**,**b** due to the almost identical coalescence temperatures (*T*_c_).


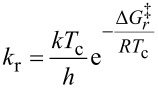



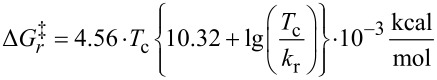



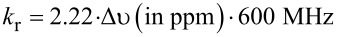


The respective Δ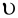 values (NH*_trans_* – NH*_cis_*) were extracted from the corresponding ^1^H NMR spectra of the specific building blocks **1a**,**b** in chlorobenzene-*d*_5_ at 25 °C (298 K). Table 3 summarizes the calculated Δ*G*^‡^_c_ values and illustrates that there is only a small difference between the calculated Δ*G*^‡^_r_ values of building blocks **1a** and **1b** in chlorbenzene-*d*_5_. It is obvious that the different saccharide moieties do not significantly influence the rotation barrier around the tertiary peptide bond. Furthermore, the calculated Δ*G*^‡^_r_ values are in good accordance with those of other PNA derivates (17.9–19 kcal/mol) [27–28].

**Table 3:** Δ*G*^‡^_c_ values of building blocks **1a**,**b**.

Entry	building block	*T*_c_ in °C (K)	Δ 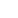 in ppm	*k*_r_ in Hz	Δ*G*^‡^_r_ in kcal/mol

1	**1a**	90–95 (363–368)	0.07	93.2	**18.1–18.3**
2	**1b**	90–95 (363–368)	0.09	119.9	**17.9–18.1**

## Conclusion

We have described the efficient chemical synthesis of a series of novel PNA-based glycopeptoids. We also studied the *cis*/*trans* rotamers of these glycopeptoids via temperature-dependent ^1^H NMR spectroscopy by determining the corresponding coalescence temperatures (*T*_c_ = 90–95 °C) and by calculating the specific rotation barrier (Δ*G*^‡^_r_) using the Eyring model. The found Δ*G*^‡^_r_ values (17.9–18.3 kcal/mol) were in good accordance with those of other known PNA-derivates (17.9–19 kcal/mol). The PNA-based glycoprotein building blocks described here will be used in combination with previously described benzoic acid-based glycopeptoids to generate glycopeptide libraries of more complex nature by automated SPOT synthesis.

## Supporting Information

File 1 Experimental data.

File 2 NMR spectra of building blocks **1a–d**, **2a–d**, **3**, **4** and **7**; 2D NMR spectra of building block **1a**.
