# Supplementary material for: Synthesis and NMR studies of malonyl-linked glycoconjugates of N-(2-aminoethyl)glycine. Building blocks for the construction of combinatorial glycopeptide libraries
Source: Beilstein J Org Chem. 2016 Aug 30;12:1939–48. doi: 10.3762/bjoc.12.183 (PMC5082468; doi:10.3762/bjoc.12.183)
Supplement: File 1 — Experimental data. [file Beilstein_J_Org_Chem-12-1939-s001.pdf]

Supporting Information

for

Synthesis and NMR studies of malonyl-linked glycoconjugates of *N*-(2-aminoethyl)glycine. Building blocks for the construction of combinatorial glycopeptide libraries

Markus Nörrlinger, Sven Hafner and Thomas Ziegler*

Address: Institute of Organic Chemistry, University of Tuebingen, Auf der Morgenstelle 18, 72076 Tuebingen, Germany

Email: Thomas Ziegler - thomas.ziegler@uni-tuebingen.de
*Corresponding author

Experimental data

General

All solvents were dried according to standard procedures, distilled and stored over molecular sieves 3 Å under an atmosphere of nitrogen prior to their use. All non-aqueous reactions were performed in oven-dried glassware under an atmosphere of nitrogen unless otherwise stated. NMR spectra were recorded on a Bruker Avance 400 spectrometer or Bruker Avance 600 spectrometer (temperature-dependent investigations) and calibrated for the solvent signal (^{1}H $CDCl_3$: 7.26 ppm; ^{13}C $CDCl_3$: 77.16 ppm; ^{1}H DMSO-d_6: 2.50 ppm; ^{13}C DMSO-d_6: 39.52 ppm; ^{1}H DMF-d_7: 8.03 ppm, 2.92 ppm, 2.75 ppm; ^{13}C DMF-d_7*:* 163.2 ppm, 34.9 ppm, 29.8 ppm*;* ^{1}H chlorobenzene-d_5: 7.14 ppm, 6.99 ppm, 6.96 ppm; ^{13}C chlorobenzene-d_5: 134.19 ppm, 129.26 ppm, 128.25 ppm, 125.96 ppm; ^{1}H-D_2O: 4.79 ppm). ESI-HRMS were

measured on a Bruker Apex II FT-ICR-MS spectometer, FAB-spectra were measured on a Finnigan model TSQ 70. Elemental analysis was performed on a HEKAtech Euro 3000 CHN analyzer. IR spectra were recorded on a Bruker Tensor 27 spectrometer. Optical rotations were measured at 589 nm (Na D-line) with a Perkin-Elmer Polarimeter 341 in a 10 cm cuvette at 20 °C. Melting points were determined with a Büchi Melting Point M-560 apparatus. Reactions were monitored by TLC on Polygram Sil G/UV silica gel plates from Machery&Nagel. Detection of spots was effected by charring with H_2SO_4 (5% in EtOH), staining by spraying the plates with an alkaline aqueous solution of potassium permanganate or by inspection of the TLC plates under UV light. Preparative chromatography was performed on silica gel (0.032–0.063 mm) from Machery&Nagel with different mixtures of solvents as eluents. All yields given below are isolated yields determined after purification of the product either by silica gel column chromatography or crystallization and were not optimized unless noted otherwise.

Starting materials

Known compounds were prepared according to literature procedures: *tert-butyl N*-[2-(*N*-9*H*-fluoren-9-ylmethoxycarbonylamino)ethyl]glycinate hydrochloride (**5**) [1], 3-oxo-3-(2,3,4,6-tetra-*O*-acetyl-β-D-glucopyranosylamino)propanoic acid (**6a**) [2], 3-oxo-3-(β-D-galactopyranosylamino)propanoic acid (**6b**) [2], 3-oxo-3-(2-acetamido-2-deoxy-3,4,6-tetra-*O*-acetyl-β-D-glucopyranosylamino)propanoic acid (**6c**) [2], 3-oxo-3-(2-acetamido-2-deoxy-3,4,6-tetra-*O*-acetyl-β-D-galactopyranosylamino)propanoic acid (**6d**) [2].

General procedure for the synthesis of compounds 1a–d

Analogous as described in [14] building blocks **1a–d** were prepared according to the following procedure.

In a 25 mL round bottom flask equipped with a gas inlet and a stirring bar, **6a–d** (1 equiv) was dissolved in 12 mL dry DMF under an atmosphere of nitrogen. The solution was cooled to 0°C and HBTU (1.5 equiv) (Method A) or EDCI·HCl (1.3 equiv) and HOBt (1.3-1.5 equiv) (Method B), and DIPEA (3.9 equiv) were added. The mixture was stirred at 0°C for 10 min. Afterwards tert-butyl *N*-[2-(*N*-9*H*-fluoren-9-yl-methoxycarbonylamino)ethyl]glycinate hydrochloride (**5**) (1 equiv) was added and the resulting solution was stirred at 0 °C for 2 h and at rt for 72 h. The solvent was removed under reduced pressure, the residue dissolved in ethyl acetate (70 mL) and successively washed with an aqueous solution of citric acid (10%) (2 × 20 mL),satd. aqueous $NaHCO_3$ solution. (3 × 20 mL), satd. aqueous NaCl solution (20 mL), dried over Na_2SO_4, filtered and concentrated. Purification of the residue by column chromatography afforded the pure title compounds **1a–d** as white amorphous solid.

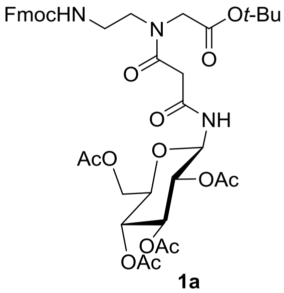

1a

***tert*-Butyl [*N*-(2,3,4,6-tetra-*O*-acetyl-β-D-glucopyranosylamino)malonyl]-*N*-2-[2-(9*H*-fluoren-9-ylmethoxycarbonylamino)ethyl]glycinate (1a)**

Method A: according to the general procedure, **6a** (100 mg, 0.23 mmol), HBTU (1.5 equiv), HOBt (1.5 equiv), DIPEA (3.9 equiv) and column chromatography (PE/EA 1:3) gave **1a** (149 mg, 79%).

Method B: according to the general procedure, **6a** (100 mg, 0.23 mmol), EDCI·HCl (1.3 equiv), HOBt (1.3 equiv), DIPEA (3.9 equiv) and column chromatography ($CHCl_3$/MeOH 200:1→50:1) gave **1a** (83 mg, 40%). R_f: 0.36 (PE/EA 1:3); $[\alpha]_D^{20}$: +0.6 (*c* 1.0, $CHCl_3$). ^{1}H-NMR ($CDCl_3$): δ 8.19, (d, 0.5H, $J_{1,NH}$ = 9.1 Hz, H-1N*H*CO *trans*-rotamer), 7.99 (d, 0.5H, $J_{1,NH}$ = 9.0 Hz, H-1N*H*CO *cis*-rotamer), 7.77-7.75 (m, 2H, H-aryl), 7.61-7.58 (m, 2H, H-aryl), 7.42-7.38 (m, 2H, H-aryl), 7.33-7.29 (m, 2H, H-aryl), 5.74, 5.45 (t, t, J_a = 5.9 Hz, J_b = 5.7 Hz, CON*H*CH_2 *cis-/trans*-rotamer), 5.28-5.22 (m, 2H, H-1, H-3), 5.09-4.97 (m, 2H, H-4, H-2), 4.44-4.39 (m, 2H, Fmoc-C*H*$_2$), 4.26-4.18 (m, 2H, H-6a, Fmoc-C*H*), 4.09-4.03 (m, 1H, H-6b), 3.98-3.89 (m, 2H, NC*H*$_2$CO$_2{}^t$Bu), 3.80-3.72 (m, 1H, H-5), 3.54-3.46 (m, 2H, NHC*H*$_2$C*H*$_2$N), 3.40-3.16 (m, 4H, COC*H*$_2$CO), 2.05, 2.04, 2.03, 2.02, 2.02, 2.01, 2.00 (8s, 12H, C*H*$_3$), 1.48, 1.47 (2s, 9H, $CO_2C(CH_3)_3$); ^{13}C NMR ($CDCl_3$): δ 170.1, 169.6, 168.9, 168.3, 168.1, 166.9, 166.6 (7C, *C*O), 156.8, 156.7 (1C, Fmoc-*CO* *cis-/trans*-rotamer), 144.0, 143.9, 141.4, 127.8, 127.8, 127.2, 125.1, 120.1 (8C, C-aryl), 83.6, 82.7 (1C, $CO_2C(CH_3)_3$ *cis-/trans*-rotamer), 78.0 (1C, C-1), 73.7, 73.6 (1C, C-5 *cis-/trans*-rotamer), 73.1, 72.9 (1C, C-3

cis-/trans-rotamer), 70.4, 70.3 (1C, C-4 *cis-/trans*-rotamer), 68.2 (1C, C-2), 66.8, 66.9 (1C, Fmoc-CH_2 *cis-/trans*-rotamer), 61.7, 60.5 (1C, C-6 *cis-/trans*-rotamer), 52.3, 50.0 (1C, N$CH_2CO_2{}^tBu$ cis-/trans-rotamer), 48.9 (1C, COCH_2CO), 47.3 (1C, Fmoc-*C*H), 40.5, 40.2, 39.0, 38.7 (2C, NHCH_2CH_2N *cis-/trans*-rotamer), 28.1, 28.1 (3C, $CO_2C(CH_3)_3$ *cis-/trans*-rotamer), 21.1, 20.8, 20.7, 20.7 (4C, CH_3); Due to the rotameric structure the signals can be exchanged. ESI-TOF-MS: Anal. Calcd. for $C_{40}H_{49}N_3O_{15}$ $[M+Na]^+$: *m/z* 834.305589; found: *m/z* 834.305244.

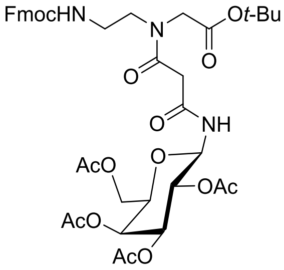

1b

tert-Butyl [_N_-(2,3,4,6-tetra-_O_-acetyl-β-D-galactopyranosylamino)malonyl]-_N_-2-[2-(9_H_-fluoren-9-ylmethoxycarbonylamino)ethyl]glycinate (1b)

Method A: according to the general procedure **6b** (100 mg, 0.23 mmol), HBTU (1.5 equiv), HOBt (1.5 equiv), DIPEA (3.9 equiv) and column chromatography (PE/EA 1:3) gave **1b** (148 mg, 79%).

Method B: according to the general procedure **6b** (100 mg, 0.23 mmol), EDCI·HCl (1.3 equiv), HOBt (1.3 equiv), DIPEA (3.9 equiv) and column chromatography ($CHCl_3$/MeOH 100:1) gave **1b** (87 mg, 47%).

R_f: 0.21 (PE/EA 1:3); $[\alpha]_D^{20}$: +7.0 (*c* 1.0, $CHCl_3$). ^{1}H-NMR ($CDCl_3$): δ 8.26 (d, 0.5H, $J_{1,NH}$ = 8.6 Hz, H-1N*H*CO *trans*-rotamer), 7.99 (d, 0.5H, $J_{1,NH}$ = 8.4 Hz, H-1N*H*CO *cis*-rotamer), 7.77-7.75 (m, 2H, H-aryl), 7.62-7.58 (m, 2H, H-aryl), 7.41-7.37 (m, 2H, H-

aryl), 7.34-7.29 (m, 2H, H-aryl), 5.76, 5.50 (t, t, 1H, J_a = 6.0 Hz, J_b = 5.5 Hz, CON*H*CH_2 *cis-/trans*-rotamer), 5.40 (dd, 1H, $J_{3,4}$ = 3.3 Hz, $J_{4,5}$ = 11.6 Hz, H-4), 5.26-5.16 (m, 2H, H-1, H-2), 5.11-5.05 (m, 1H, H-3), 4.44-4.39 (m, 2H, Fmoc-CH_2), 4.23-4.19 (m, 1H, Fmoc-C*H*), 4.13-3.90 (m, 5H, H-6a, H-6b, NC$H_2$$CO_2$^{t}Bu, H-5), 3.56-2.83 (m, 6H, NHCH_2CH_2N, COCH_2CO), 2.14, 2.14, 2.13, 2.12, 2.10, 2.06, 2.02, 1.99, 1.98, 1.97 (10s, 12H, CH_3), 1.48, 1.47 (2s, 9H, CO_2C(CH_3)$_3$); ^{13}C NMR ($CDCl_3$): δ 170.8, 170.6, 170.5, 170.3, 170.2, 170.0, 169.1, 168.9, 168.3, 168.2, 166.6, 166.4 (12C, *C*O), 156.8, 156.7 (2C, Fmoc-*C*O *cis-/trans*-rotamer), 144.1, 144.0, 143.9, 141.4, 127.9, 127.8, 127.2, 127.1, 125.2, 125.1, 120.1 (11C, C-aryl), 83.7, 82.8 (1C, CO_2*C*(CH_3)$_3$ *cis-/trans*-rotamer b), 78.4 (1C, C-1), 72.5, 72.4 (1C, C-5 *cis-/trans*-rotamer), 71.3, 71.2 (1C, C-3 *cis-/trans*-rotamer), 68.2, 68.1 (1C, C-2 *cis-/trans*-rotamer), 67.3, 67.2 (1C, C-4 *cis-/trans*-rotamer), 67.0, 66.8 (1C, Fmoc-*C*H_2 *cis-/trans*-rotamer), 61.4 (1C, C-6), 52.4, 49.2 (1C, N*C*$H_2$$CO_2$^{t}Bu *cis-/trans*-rotamer), 49.2 (1C, CO*C*H_2CO), 47.4, 47.3 (1C, Fmoc-*C*H *cis-/trans*-rotamer), 40.4, 40.2, 39.2, 39.6 (2C, NH*C*$H_2$$CH_2$N *cis-/trans*-rotamer), 28.2, 28.1 (3C, CO_2C(*C*H_3)$_3$ *cis-/trans*-rotamer), 20.8, 20.8, 20.7, 20.7 (4C, *C*H_3); Due to the rotameric structure the signals can be exchanged. FT-ICR-MS: Anal. Calcd. for $C_{40}H_{49}N_3O_{15}$ $[M+Na]^+$: *m/z* 834.305589; found: *m/z* 834.304890.

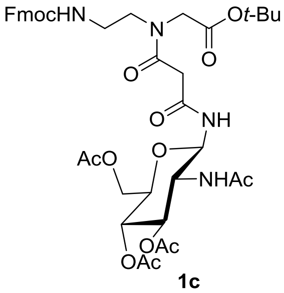

1c

***tert*-Butyl [*N*-(2-acetamido-3,4,6-tri-*O*-acetyl-2-deoxy-β-D-glucopyranosylamino)malonyl]-*N*-2-[2-(9*H*-fluoren-9-ylmethoxycarbonylamino)ethyl]glycinate (1c)**

Method A: according to the general procedure **6c** (100 mg, 0.23 mmol), HBTU (1.5 equiv), HOBt (1.5 equiv), DIPEA (3.9 equiv) and column chromatography ($CHCl_3$/MeOH 100:1→50:1) gave **1c** (141 mg, 75%).

Method B: according to the General Procedure **6c** (100 mg, 0.23 mmol), EDCI·HCl (1.3 equiv), HOBt (1.3 equiv), DIPEA (3.9 equiv) and column chromatography ($CHCl_3$/MeOH 100:1→50:1) gave **1c** (106 mg, 57%).

R_f: 0.61 ($CHCl_3$/MeOH 25:1); $[\alpha]_D^{20}$: -0.7 (*c* 1.0, $CHCl_3$). ^{1}H-NMR ($CDCl_3$): δ

7.82 (d, 0.5H, $J_{1,NH}$ = 8.4 Hz, H-1N*H*COCH_2 *trans*-rotamer), 7.76-7.74 (m, 2H, H-aryl), 7.64-7.58 (m, 2H, H-aryl), 7.53 (d, 1H, $J_{1,NH}$ = 8.3 Hz, H-1N*H*COCH_2 *cis*-rotamer), 7.41-7.37 (m, 2H, H-aryl), 7.32-7.28 (m, 2H, H-aryl), 7.11, 6.54-6.50 (d, m, 1H, *J* = 8.8 Hz, N*H* *cis-/trans*-rotamer), 6.54-6.50, 5.99 (m, t, 1H, *J* = 5.4 Hz, CON*H*CH_2 *cis-/trans*-rotamer), 5.49, 5.28-5.20 (t, m, 1H, *J* = 9.9 Hz, H-3 *cis-/trans*-rotamer), 5.39 (t, 0.5H, *J* = 9.0 Hz, H-1 *cis*-rotamer), 5.28-5.20 (m, 1H, H-1 *trans*-rotamer), 5.07, 4.96 (t, t, 1H, J_a = 9.7 Hz, J_b = 9.5 Hz, H-4 *cis-/trans*-rotamer), 4.63-4.35 (m, 2H, Fmoc-C*H_2*), 4.27-3.99 (m, 5H, Fmoc-C*H*, H-6a, H-2, H-6b, H-5), 3.89-3.80 (m, 2H, NC*H_2*CO_2^{t}Bu), 3.55-2.96 (m, 6H, NHC*H_2*C*H_2*, COC*H_2*CO), 2.05, 2.00, 1.97, 1.96, 1.93, 1.91, 1.90 (7s, 12H, C*H_3*), 1.47, 1.43 (2s, 9H, $CO_2C(CH_3)_3$); ^{13}C NMR ($CDCl_3$):

δ 173.2, 172.4, 171.5, 171.2, 170.8, 169.6, 169.5, 168.8, 168.5, 168.3, 168.1, 167.3, 167.1, 156.8 (14C, *C*O), 144.0, 143.8, 141.4, 127.9, 127.8, 127.2, 127.1, 125.3, 125.2, 120.1, 120.0 (11C, C-aryl), 83.5, 82.4 (1C, $CO_2C(CH_3)_3$ cis-/trans-rotamer), 80.1, 79.9 (1C, C-1 *cis-/trans*-rotamer), 73.4, 73.0 (1C, C-5 *cis-/trans*-rotamer), 72.7, 72.3 (1C, C-3 *cis-/trans*-rotamer), 68.7, 68.3 (1C, C-4 *cis-/trans*-rotamer), 67.1, 66.6 (1C, Fmoc-CH_2 *cis-/trans*-rotamer), 62.0, 61.9 (1C, C-6 *cis-/trans*-rotamer), 53.0, 52.6 (1C, C-2 *cis-/trans*-rotamer), 49.9, 49.6 (1C, N$CH_2CO_2{}^tBu$ *cis-/trans*-rotamer), 47.4, 47.3 (1C, Fmoc-*C*H *cis-/trans*-rotamer), 41.8, 41.7 (1C, COCH_2CO *cis-/trans*-rotamer), 39.6, 39.0 (2C, NHCH_2CH_2), 28.1 (3C, $CO_2C(CH_3)_3$), 23.0, 22.9, 20.8, 20.7, 20.6 (5C, CH_3); Due to the rotameric structure the signals can be exchanged. FT-ICR-MS: Calcd. for $C_{40}H_{50}N_4O_{14}$ $[M+Na]^+$: *m/z* 833.321573; found: *m/z* 833.321147.

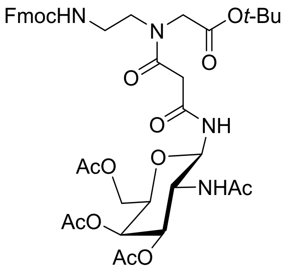

1d

***tert*-Butyl [*N*-(2-acetamido-3,4,6-tri-*O*-acetyl-2-deoxy-β-D-galactopyranosylamino)malonylamino]-*N*-2-[2-(9*H*-fluoren-9-ylmethoxycarbonylamino)ethyl]glycinate (1d)**

Method A: according to the general procedure **6d** (100 mg, 0.23 mmol), HBTU (1.5 equiv), HOBt (1.5 equiv), DIPEA (3.9 equiv) and column chromatography ($CHCl_3$/MeOH 100:1→50:1) gave **1d** (145 mg, 77%).

Method B: according to the general procedure **6d** (100 mg, 0.23 mmol), EDCI·HCl (1.3 equiv), HOBt (1.3 equiv), DIPEA (3.9 equiv) and column chromatography ($CHCl_3$/MeOH 100:1→50:1) gave **1d** (78 mg, 42%).

R_f: 0.50 ($CHCl_3$/MeOH 25:1); $[\alpha]_D^{20}$: -5.7 (*c* 1.0, $CHCl_3$). ^{1}H-NMR ($CDCl_3$): δ 7.87 (d, 0.5H, $J_{1,NH}$= 8.3 Hz, H-1N*H*COCH_2 *trans*-rotamer), 7.76-7.73 (m, 2H, H-aryl), 7.71 (d, 0.5H, $J_{1,NH}$ = 7.4 Hz, H-1N*H*COCH_2 *cis*-rotamer), 7.61-7.56 (m, 2H, H-aryl), 7.40-7.36 (m, 2H, H-aryl), 7.33-7.30 (m, 2H, H-aryl), 7.33-7.30, 6.64 (m, d, 1H, J_b = 8.9 Hz, N*H* *cis-/trans*-rotamer), 6.73, 6.09 (s, s, 1H, CON*H*CH_2 *cis-/trans*-rotamer), 5.58 (dd, 1H, $J_{3,4}$ = 2.7 Hz, $J_{4,5}$ = 11.0 Hz, H-4), 5.43 (t, 0.5H, *J* = 8.8 Hz, H-1 *cis*-rotamer), 5.36-5.25 (m, 1H, H-3), 5.21 (t, 0.5H, *J* = 9.9 Hz, H-1 *trans*-rotamer), 4.64-2.88 (m, 15H, Fmoc-CH_2, Fmoc-C*H*, H-6a, H-2, H-6b, H-5, NCH_2CO_2^{t}Bu, NHCH_2CH_2N, COCH_2CO), 2.11, 1.98, 1.96, 1.92, 1.90, 1.78 (6s, 12H, CH_3), 1.47, 1.43 (2s, 9H, $CO_2C(CH_3)_3$); ^{13}C NMR ($CDCl_3$): δ 173.7, 171.0, 170.8, 170.5, 170.4, 170.3, 169.0, 168.9, 168.3, 167.7, 167.3, 167.1, 156.9 (13C, *C*O), 144.5, 144.0, 143.6, 141.4, 141.3, 127.9, 127.8, 127.3, 127.2, 125.6, 125.2, 120.1, 120.0, (13C, C-aryl), 83.6, 82.4 (1C, $CO_2C(CH_3)_3$ *cis-/trans*-rotamer), 80.6, 80.4 (1C, C-1 *cis-/trans*-rotamer), 72.2, 71.9 (1C, C-5 *cis-/trans*-rotamer), 70.4, 69.8 (1C, C-4 *cis-/trans*-rotamer), 67.4, 66.9 (1C, Fmoc-*C*H_2 *cis-/trans*-rotamer), 66.8, 66.7 (1C, C-3 *cis-/trans*-rotamer), 61.5, 61.4 (1C, C-6 *cis-/trans*-rotamer), 49.7, 49.6 (2C, NH*C*H_2*C*H_2N), 49.4, 49.2 (1C, C-2 *cis-/trans*-rotamer), 47.4, 47.0 (1C, Fmoc-*C*H *cis-/trans*-rotamer), 41.7 (1C, CO*C*H_2CO), 39.5 (1C, N*C*H_2CO_2^{t}Bu), 28.1 (3C, $CO_2C(CH_3)_3$), 23.1, 20.9, 20.8, 20.7, 20.3 (5C, *C*H_3); Due to the rotameric structure the signals can be exchanged. FT-ICR-MS: Calcd. for $C_{40}H_{50}N_4O_{14}$ $[M+Na]^+$: *m/z* 833.321573; found: *m/z* 833.321729.

General procedure for *tert*-butyl ester hydrolysis

In almost the same way as described in [14] building blocks **1a-d** were converted into their free carbon acids **2a-d** according to following procedure.

Compounds **1a–d** (1 equiv) were dissolved in a mixture of formic acid and DCM (2:1) and stirred at rt for 38 h. DCM and formic acid were removed by passing a stream of N_2 through the solution. The residue was repeatedly dissolved in toluene and concentrated in vacuo (5 × 20 mL) in order to remove remaining traces of formic acid. Chromatography of the residue afforded compounds **2a–d** as white amorphous solids.

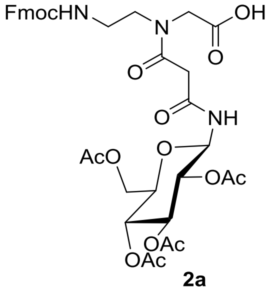

2a

[*N*-(2,3,4,6-Tetra-*O*-acetyl-β-D-glucopyranosylamino)malonyl]-*N*-2-[2-(9*H*-fluoren-9-ylmethoxycarbonylamino)ethyl]glycine (2a)

According to the general procedure, **1a** (294 mg, 0.36 mmol) in 12 mL HCO_2H/DCM afforded after column chromatography ($CHCl_3$/MeOH 50:1 + 1 % HCO_2H→$CHCl_3$/MeOH 25:1 + 1 % HCO_2H) **2a** (269 mg, 98%).

R_f: 0.52 ($CHCl_3$/MeOH 25:1 + 1 % HCO_2H); $[\alpha]_D^{20}$: -2.0 (*c* 1.0, $CHCl_3$); ^{1}H-NMR (DMSO-d_6) δ 12.89 (s, 1H, CO_2H), 8.84 (t, 1H, $J_{1,NH}$ = 10.3 Hz, H-1N*H*CO), 7.89-7.88 (m, 2H, H-aryl), 7.68-7.66 (m, 2H, H-aryl), 7.41 (t, 2H, *J* = 7.4 Hz, H-aryl), 7.35-7.31 (m, 3H, H-aryl, N*H*), 5.42-5.32 (m, 2H, H-1, H-3), 4.89 (t, 1H, $J_{3,4}$ = 9.6 Hz, H-4),

4.84-4.77 (m, 1H, H-2), 4.32-4.27 (m, 2H, Fmoc-C*H*$_2$), 4.21 (t, 1H, J = 6.4 Hz, Fmoc-C*H*), 4.16-4.07 (m, 3H, H-6a, H-6b, H-5), 3.98-3.89 (m, 2H, C*H*$_2$CO$_2$H), 3.48-3.10 (m, 6H, NHC*H*$_2$C*H*$_2$, COC*H*$_2$CO), 1.99, 1.97, 1.92 (3s, 12H, C*H*$_3$); ^{13}C-NMR (DMSO-d$_6$) δ 170.8, 170.1, 169.5, 169.4, 169.3, 169.2, 167.7, 167.2, 167.0, 166.8 (10C, C=O), 156.3, 156.1 (1C, Fmoc-C=O *cis-/trans*-rotamer), 143.9, 143.9, 140.7, 140.7, 127.7, 127.1, 125.2, 125.1, 120.2 (9C, C-aryl), 76.8 (C-1), 72.8 (C-3), 72.2 (C-5), 70.5 (C-2), 67.9 (C-4), 65.5 (1C, Fmoc-*C*H$_2$), 61.8 (C-6), 48.1 (1C, *C*H$_2$CO$_2$H), 47.8 (1C, CO*C*H$_2$CO), 46.7 (1C, Fmoc-*C*H), 40.8 (1C, NH*C*H$_2$CH$_2$), 20.5, 20.4, 20.4, 20.3 (4C, *C*H$_3$); Due to the rotameric structure the signals can be exchanged. FT-ICR-MS: Calcd. for $C_{35}H_{41}N_3O_{15}Na$ [M+Na]$^+$: *m/z* 778.242989; found.: *m/z* 778.242857.

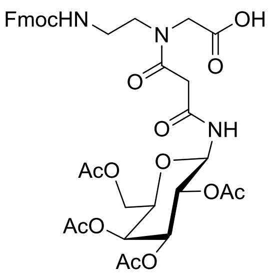

2b

[*N*-(2,3,4,6-Tetra-*O*-acetyl-β-D-galactopyranosylamino)malonyl]-*N*-2-[2-(9*H*-fluoren-9-ylmethoxycarbonylamino)ethyl]glycine (2b)

According to the general procedure, **1b** (243 mg, 0.30 mmol) in 12 mL HCO$_2$H/DCM afforded after column chromatography (CHCl$_3$/MeOH 50:1 + 1 % HCO$_2$H→CHCl$_3$/MeOH 25:1 + 1 % HCO$_2$H) **2b** (219 mg, 97%).

R$_f$: 0.50 (CHCl$_3$/MeOH 25:1 + 1 % HCO$_2$H); $[\alpha]_D^{20}$: $[\alpha]_D^{20}$: +6.3 (*c* 1.0, CHCl$_3$); ^{1}H-NMR (DMSO-d$_6$) δ 12.92 (s, 1H, CO$_2$*H*), 8.89 (t, 1H, $J_{1,NH}$ = 8.5 Hz, H-1N*H*CO), 7.89-7.87 (m, 2H, H-aryl), 7.68-7.66 (m, 2H, H-aryl), 7.41 (t, 1H, *J* = 7.4 Hz, H-aryl), 7.35-7.31 (m, 3H, H-aryl, N*H*), 5.37-5.28 (m, 3H, H-1, H-4, H-3), 5.03-4.99 (m, 1H, H-2),

4.32-4.22 (m, 4H, Fmoc-C*H*$_2$, H-5, Fmoc-C*H*), 4.06-3.94 (m, 4H, H-6a, H-6b, C*H*$_2$CO$_2$H), 3.36-3.09 (m, 6H, NHC*H*$_2$C*H*$_2$, COC*H*$_2$CO), 2.09, 1.99, 1.98, 1.96, 1.91 (5s, 12H, C*H*$_3$); ^{13}C-NMR (DMSO-d$_6$) δ 170.7, 169.9, 169.9, 169.5, 169.4, 167.8, 167.3, 167.0, 166.9 (9C, C=O), 156.3, 156.1 (1C, Fmoc-C=O *cis-/trans*-rotamer), 143.9, 143.9, 140.8, 140.7, 127.7, 127.1, 125.2, 125.2, 120.2 (9C, C-aryl), 77.2 (C-1), 71.4 (C-5), 70.8 (C-3), 68.1 (C-2), 67.6 (C-4), 65.5 (1C, Fmoc-*C*H$_2$), 61.5 (C-6), 48.1 (1C, *C*H$_2$CO$_2$H), 47.8 (1C, CO*C*H$_2$CO), 46.7 (1C, Fmoc-*C*H), 41.2, 40.7 (2C, NH*C*H$_2$*C*H$_2$), 20.5, 20.5, 20.4, 20.4 (4C, *C*H$_3$); Due to the rotameric structure the signals can be exchanged. FT-ICR-MS: Calcd. for $C_{35}H_{41}N_3O_{15}Na$ $[M+Na]^+$: *m/z* 778.242989; found.: *m/z* 778.243134.

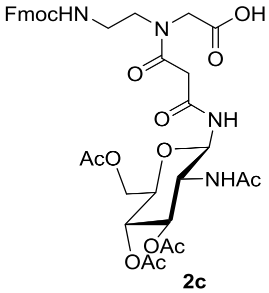

2c

[*N*-(2-acetamido-3,4,6-tri-*O*-acetyl-2-deoxy-β-D-glucopyranosylamino)malonyl]-*N*-2-[2-(9*H*-fluoren-9-ylmethoxycarbonylamino)ethyl]glycine (2c)

According to the general procedure, **1c** (273 mg, 0.34 mmol) in 12 mL HCO$_2$H/DCM afforded after column chromatography (CHCl$_3$/MeOH 50:1 + 1 % HCO$_2$H→CHCl$_3$/MeOH 25:1 + 1 % HCO$_2$H) **2c** (245 mg, 97%).

R$_f$: 0.28 (CHCl$_3$/MeOH 25:1 + 1 % HCO$_2$H); $[\alpha]_D^{20}$: +0.7 (*c* 1.0, CHCl$_3$); ^{1}H-NMR (DMSO-d$_6$) δ 12.84 (s, 1H, CO$_2$*H*), 8.77-8.73 (m, 1H, H-1N*H*CO *cis-/trans*-rotamer), 7.95-7.92 (m, 1H, N*H*), 7.89-7.87 (m, 2H, H-aryl), 7.68-7.66 (m, 2H, H-aryl), 7.43-7.39 (m, 2H, H-aryl), 7.35-7.31 (m, 3H, H-aryl, CON*H*CH$_2$), 5.22-5.09 (m, 2H, H-1, H-

3), 4.82 (t, 1H, $J_{3,4}$ = 9.8 Hz, H-4), 4.32-4.26 (m, 2H, Fmoc-CH_2), 4.23-4.14 (m, 2H, Fmoc-C*H* *cis-/trans*-rotamer, H-6a), 4.04-3.81 (m, 5H, H-2, H-6b, H-5, COCH_2CO), 3.42-3.26 (m, 4H, CH_2COH, NHCH$_2$CH_2), 3.22-3.07 (m, 2H, NHCH_2CH$_2$), 1.99, 1.98, 1.96, 1.90, 1.76, 1.73 (6s, 12H, CH_3); ^{13}C-NMR (DMSO-d$_6$) δ 171.3, 170.9, 170.1, 169.7, 169.6, 169.4, 167.7, 167.2, 167.0, 166.9 (10C, C=O), 156.3, 156.1 (1C, Fmoc-C=O *cis-/trans*-rotamer), 143.9, 143.8, 140.8, 140.7, 127.7, 127.1, 125.3, 125.1, 120.2 (9C, C-aryl), 78.0, 78.0 (1C, C-1 *cis-/trans*-rotamer), 73.3 (C-3), 72.3 (C-5), 68.4 (C-4), 65.5 (1C, Fmoc-*C*H$_2$), 61.8 (C-6), 52.1, 52.0 (1C, C-2 *cis-/trans*-rotamer), 50.9 (1C, CO*C*H$_2$CO), 48.1, (1C, *C*H$_2$CO$_2$H), 47.8, 46.7 (1C, Fmoc-*C*H cis-/trans-rotamer), 41.2, 40.8 (2C, NH*C*H$_2$*C*H$_2$), 22.6, 20.6, 20.4, 20.4 (4C, CH$_3$); Due to the rotameric structure the signals can be exchanged. FT-ICR-MS: Calcd. for $C_{36}H_{42}N_4O_{14}Na$ [M+Na]$^+$: *m/z* 777.258973; found: *m/z* 777.258435.

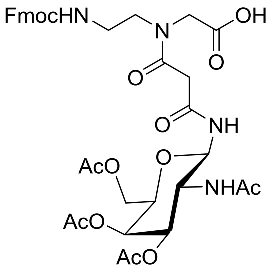

2d

[*N*-(2-acetamido-3,4,6-tri-*O*-acetyl-2-deoxy-β-D-galactopyranosylamino)malonyl]-*N*-2-[2-(9*H*-fluoren-9-ylmethoxycarbonylamino)ethyl]glycine (2d)

According to the General Procedure **1d** (124 mg, 0.15 mmol) in 6 mL HCO$_2$H/DCM afforded after column chromatography (CHCl$_3$/MeOH 50:1 + 1 % HCO$_2$H→CHCl$_3$/MeOH 10:1 + 1 % HCO$_2$H) **2d** (111 mg, 97%).

R_f: 0.23 ($CHCl_3$/MeOH 25:1 + 1 % HCO_2H); $[\alpha]_D^{20}$: +1.3 (*c* 1.0, $CHCl_3$); ^{1}H-NMR (DMSO-d_6) δ 12.88 (s, 1H, CO_2H), 8.69-8.67 (m, 1H, H-1N*H*CO *cis-/trans*-rotamer), 7.92-7.88 (m, 3H, N*H*, H-aryl), 7.69-7.66 (m, 2H, H-aryl), 7.43-7.31 (m, 5H, H-aryl, CON*H*CH_2), 5.26-5.25 (m, 1H, H-3), 5.13-5.07 (m, 1H, H-1), 5.05-5.01 (m, 1H, H-4), 4.32-4.22 (m, 3H, Fmoc-CH_2, Fmoc-CH), 4.06-3.91 (m, 6H, H-5, H-6a, H-6b, H-2, COCH_2CO), 3.35-3.10 (m, 6H, NHCH_2CH_2, C$H_2$$CO_2$H), 2.09, 2.08, 1.98, 1.97, 1.89, 1.78, 1.75 (7s, 12H, CH_3); ^{13}C-NMR (DMSO-d_6) δ 170.1, 170.0, 169.6, 167.8, 167.8, 167.2, 167.1, 166.9 (8C, C=O), 156.3, 156.1 (1C, Fmoc-C=O, *cis-/trans*-rotamer), 143.9, 143.9, 140.8, 140.7, 127.7, 127.1, 120.2, 120.1 (8C, C-aryl), 78.8, 78.7 (1C, C-1 *cis-/trans*-rotamer), 71.4 (C-5), 70.8 (C-4), 66.7 (C-3), 65.5 (1C, Fmoc-*C*H_2), 61.6 (C-6), 51.7 (C-2), 48.3 (1C, CO*C*H_2CO), 48.2 (*C*$H_2$$CO_2$H), 46.7 (1C, Fmoc-*C*H), 40.7 (2C, NH*C*H_2*C*H_2), 22.7, 20.6, 20.5, 20.5 (4C, *C*H_3); Due to the rotameric structure the signals can be exchanged. ESI-TOF-MS: Calcd. for $C_{36}H_{41}N_4O_{14}$ $[M-H]^-$: *m/z* 753.26248; found: *m/z* 753.26314.

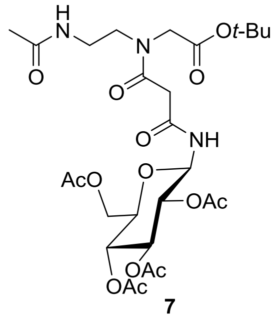

7

***tert*-Butyl [*N*-(2,3,4,6-tetra-*O*-acetyl-β-D-glucopyranosylamino)malonyl]-*N*-2-[2-(acetylamino)ethyl]glycinate (7)**

Compound **1a** (105 mg, 0.13 mmol) was dissolved in a 1:1 mixture of Et_3N/DMF (6 mL) and the mixture was stirred for 3 ½ h at rt until TLC indicated complete

consumption of the starting material. Ac_2O (4 mL) was added and the mixture was stirred at rt for 30 min. The solvent was removed under reduced pressure, the residue dissolved in ethyl acetate (70 mL) and successively washed with an aqueous solution of citric acid (10%) (2 × 20 mL),satd. aqueous $NaHCO_3$ solution. (3 × 20 mL), satd. aqueous NaCl solution (20 mL), dried over Na_2SO_4, filtered and concentrated. Purification of the residue by column chromatography ($CHCl_3$/MeOH 100:1→50:1) afforded the pure title compound **7** (49 mg, 60%) as amorphous solid.

R_f: 0.26 ($CHCl_3$/MeOH 50:1); $[\alpha]_D^{20}$: -2.0 (*c* 1.0, $CHCl_3$); ^{1}H-NMR ($CDCl_3$) δ 8.19 (d, 0.5H, $J_{1,NH}$ = 9.1 Hz, H-1N*H*$COCH_2$ *trans*-rotamer), 7.99 (d, 0.5H, $J_{1,NH}$ = 9.1 Hz, H-1N*H*$COCH_2$ *cis*-rotamer), 6.73 (t, 0.5H, *J* = 5.7 Hz, N*H* *cis*-rotamer), 6.42 (t, 0.5H, *J* = 5.1 Hz, N*H* *trans*-rotamer), 5.28-5.21 (m, 2H, H-1, H-3,), 5.06-4.94 (m, 2H, H-2, H-4), 4.25-4.19 (m, 1H, H-6a,), 4.07-4.01 (m, 2H, H-6b,), 3.96-3.85 (m, 2H, $NCH_2CO_2{}^tBu$ *cis-/trans*-rotamer), 3.79-3.76 (m, 1H, H-5), 3.56-3.11 (m, 6H, $NHCH_2CH_2$, $COCH_2CO$ *cis-/trans*-rotamer), 2.04, 2.03, 2.01, 1.99, 1.98, 1.97, 1.95, 1.92 (10s, 12H, CH_3), 1.44 (s, 9H, $CO_2C(CH_3)_3$); ^{13}C-NMR ($CDCl_3$) δ 170.9, 170.8, 170.7, 170.7, 170.4, 170.0, 170.0, 169.6, 169.2, 169.1, 168.4, 168.0, 167.0, 166.6 (8C, C=O *cis-/trans*-rotamer), 83.7, 82.7 (1C, $CO_2C(CH_3)_3$ *cis-/trans*-rotamer), 78.0, 78.0 (1C, C-1 *cis-/trans*-rotamer), 73.7 (C-5), 73.1, 73.0 (1C, C-3 *cis-/trans*-rotamer), 70.4, 70.3 (1C, C-4 *cis-/trans*-rotamer), 68.2 (C-2), 61.8 (C-6), 52.0, 49.9 (1C, $NCH_2CO_2{}^tBu$ *cis-/trans*-rotamer), 49.7, 48.5 (1C, $NHCH_2CH_2N$ *cis-/trans*-rotamer), 40.4, 40.2 (1C, $COCH_2CO$ *cis-/trans*-rotamer), 37.8, 37.7 ($NHCH_2CH_2N$ *cis-/trans*-rotamer), 28.0, 28.0 (3C, $CO_2C(CH_3)_3$ *cis-/trans*-rotamer), 23.2, 23.1, 20.8, 20.7, 20.6 (5C, CH_3); Due to the rotameric structure the signals can be exchanged. ESI-TOF-MS: Calcd. for $C_{27}H_{41}N_3O_{14}Na$ $[M+Na]^+$: *m/z* 654.24807; found: *m/z* 654.24871.

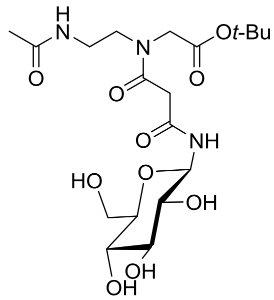

3

tert-Butyl [_N_-(β-D-glucopyranosylamino)malonyl]-_N_-2-[2-(acetylaminoethyl)glycinate (3)

Compound **7** (46 mg, 0.07 mmol) was dissolved in an 6:1 mixture of MeOH/NH_3 in MeOH (7 N) (7 mL) for 1 h at rt until the TLC indicated complete consumption of the starting material. The solvent was removed under reduced pressure to afforded the pure title compound **3** (34 mg, 100%) as amorphous solid.

R_f: 0.41 ($CHCl_3$/MeOH 2:1); $[\alpha]_D^{20}$: -2.0 (*c* 1.0, H_2O); ^{1}H-NMR (D_2O) δ 5.01-4.97 (m, 1H, H-1 *cis-/trans*-rotamer), 4.25 (s, 1H, N$CH_2CO_2{}^t$Bu *trans*-rotamer), 4.06 (s, 1H, N$CH_2CO_2{}^t$Bu *cis*-rotamer), 3.89 (dd, 1H, $J_{5,6a}$ = 1.9 Hz, $J_{6a,6b}$ = 12.3 Hz, H-6a), 3.73 (dd, 1H, $J_{5,6b}$ = 5.3 Hz, $J_{6a,6b}$ = 12.3 Hz, H-6b), 3.68-3.35 (m, 10H, COCH_2CO, NHCH_2CH_2N, H-2, H-3, H-4, H-5), 2.01 (s, 2H, CH_3 *cis*-rotamer), 1.98 (s, 1H, CH_3 *trans*-rotamer), 1.50 (s, 3H, $CO_2C(CH_3)_3$ *trans*-rotamer), 1.48 (s, 6H, $CO_2C(CH_3)_3$ *cis*-rotamer); ^{13}C-NMR (D_2O) δ 175.3, 175.1, 171.0, 170.7, 170.6, 170.6, 170.4 (7C, C=O cis-/trans-rotamer), 85.5, 84.8 (1C, $CO_2C(CH_3)_3$ *cis-/trans*-rotamer), 80.1 (C-1), 78.3 (C-5), 77.1 (C-3), 72.5 (C-4), 69.9 (C-2), 61.2 (C-6), 52.2 (1C, N$CH_2CO_2{}^t$Bu *trans*-rotamer), 50.6 (1C, N$CH_2CO_2{}^t$Bu *cis*-rotamer), 49.6, 47.4 (1C, NHCH_2CH_2N cis-/trans-rotamer), 42.1 (1C, COCH_2CO *trans*-rotamer), 41.6 (1C, COCH_2CO *cis*-rotamer), 38.2, 37.6 (1C, NHCH_2CH_2N *cis-/trans*-rotamer), 27.9 (3C, $CO_2C(CH_3)_3$), 22.6 (1C, CH_3); Due to the rotameric structure the signals can be exchanged. ESI-TOF-MS: Calcd. for $C_{19}H_{33}N_3O_{10}Na$ $[M+Na]^+$: *m/z* 486.20581 found: *m/z* 486.20602.

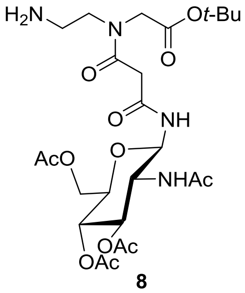

8

tert-Butyl [_N_-(2-acetamido-3,4,6-tri-_O_-acetyl-2-deoxy-β-D-glucopyranosylamino)malonyl]-_N_-2-[2-(aminoethyl)glycinate (8)

In a similar manner as described in [14] building block **1c** was deprotected according to following procedure.

The Fmoc-protected amine **1c** (133 mg, 0.16 mmol) was stirred in 6 mL 20% piperidine/DMF at rt for 3 ½ h until TLC indicated complete consumption of the starting material. The solvent was removed under reduced pressure and the residue was co-evaporated with toluene (5 × 20 mL) to afford crude title compound **8** which was used without further purification.

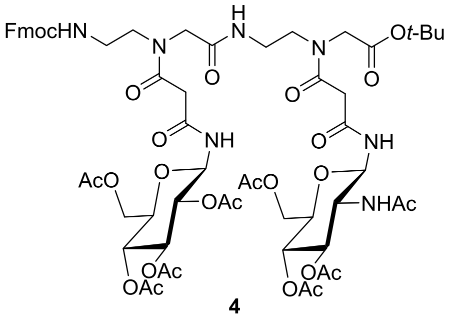

4

tert-Butyl 3-[_N_-(2-acetamido-3,4,6-tri-_O_-acetyl-2-deoxy-β-D-glucopyranosylamino)malonyl]-11-(9_H_-fluoren-9-ylmethoxycarbonyl)-9-[_N_-(2,3,4,6-tetra-_O_-acetyl-β-D-glucopyranosyl)malonyl]-3,6,9,12-tetraazadodecanoate (4)

Analogous as described in [14] dimeric glycoconjugate **4** was prepared according to following procedure.

In a 25 mL round bottom flask equipped with a gas inlet and a stirring bar **2a** (124 mg, 0.16 mmol) was dissolved in 12 mL dry DMF under an atmosphere of nitrogen. The solution was cooled to 0 °C and HBTU (1.3 equiv), HOBt (1.3 equiv) and DIPEA (3.9 equiv) were added. The mixture was stirred at 0 °C for 10 min. Afterwards **8** (1 equiv) was added and the resulting solution was stirred at 0 °C for 2 h. Thereafter the solution was stirred at rt for 14 h. The solvent was removed under reduced pressure, the residue dissolved in ethyl acetate (70 mL) and successively washed with an aqueous solution of citric acid (10%) (2 × 20 mL),satd. aqueous $NaHCO_3$ solution. (3 × 20 mL), satd. aqueous NaCl solution (20 mL), dried over Na_2SO_4, filtered and concentrated. Purification of the residue by column chromatography ($CHCl_3$/MeOH 100:1→25:1) afforded pure title compound **4** (126 mg, 58%) as amorphous solid.

R_f: 0.27 ($CHCl_3$/MeOH 25:1); $[\alpha]_D^{20}$: -8.3 (*c* 1.0, $CHCl_3$); ^{1}H-NMR ($CDCl_3$) δ 8.77, 8.39, 8.32, 8.21, 8.11, 7.98, 7.71, 7.53, 7.12, 6.95, 6.75, 6.17, 5.75 (d, d, d, d, d, m, d, d, d, d, d, s, s, 4H, H-1N*H*, H-1'N*H*, CON*H*CH_2, H-2'N*H* *trans-/trans-*, *trans-/cis-*, *cis-/trans-*, *cis-/cis*-rotamer), 7.77-7.75 (m, 2H, H-aryl), 7.61-7.59 (m, 2H, H-aryl), 7.41-7.37 (m, 2H, H-aryl), 7.33-7.29 (m, 2H, H-aryl), 5.38-5.19 (m, 4 H, H-1, H-1', H-3, H-3' *trans-/trans-*, *trans-/cis-*, *cis-/trans-*, *cis-/cis*-rotamer), 5.13-4.99 (m, 3H, H-4', H-4, H-2 *trans-/trans-*, *trans-/cis-*, *cis-/trans-*, *cis-/cis*-rotamer), 4.41-3.01 (m, 26H, Fmoc-C*H*$_2$, Fmoc-C*H*, H-6a, H-6b, H-6a', H-6b', H-5, H-5', H-2', NC*H*$_2$'CO2tBu, $NHCH_2$'CH_2'N, NCH_2CON, $NHCH_2CH_2N$, $COCH_2CO$, $CHCH_2$'CO *trans-/trans-*, *trans-/cis-*, *cis-/trans-*, *cis-/cis*-rotamer), 2.05, 2.05, 2.05, 2.03, 2.02, 2.01, 2.00, 1.99, 1.95, 1.94, 1.93, 1.92 (12s, 24H, C*H*$_3$ *trans-/trans-*, *trans-/cis-*, *cis-/trans-*, *cis-/cis*-rotamer), 1.47, 1.46, 1.44, 1.42 (4s, 9H, $CO_2C(CH_3)_3$ *trans-/trans-*, *trans-/cis-*, *cis-/trans-*, *cis-*

/*cis*-rotamer); ^{13}C-NMR ($CDCl_3$) δ 171.0, 170.8, 170.7, 170.3, 170.2, 169.7, 169.6, 169.3, 168.9, 168.7, 168.4, 168.4, 168.2, 167.8, 167.5, 167.3, 156.9, 156.8 (15C, C=O rotamer a, b, c, d), 144.1, 144.1, 144.1, 144.0, 144.0, 141.4, 127.9, 127.2, 125.2, 120.1 (10C, C-aryl *trans-/trans-*, *trans-/cis-*, *cis-/trans-*, *cis-/cis*-rotamer), 84.0, 83.5, 83.3, 82.7 (1C, $CO_2C(CH_3)_3$ *trans-/trans-*, *trans-/cis-*, *cis-/trans-*, *cis-/cis*-rotamer), 79.6, 79.6, 79.0, 78.1, 78.1 (2C, C-1, C-1' *trans-/trans-*, *trans-/cis-*, *cis-/trans-*, *cis-/cis*-rotamer), 73.8, 73.7, 73.6, 73.5, 73.4, 73.2, 73.1, 73.0 (2C, C-5, C-5' rotamer a, b, c, d), 70.7, 70.5 (1C, C-4 *trans-/trans-*, *trans-/cis-*, *cis-/trans-*, *cis-/cis*-rotamer), 68.5, 68.5, 68.3, 68.2, 68.2 (2C, C-2, C-4' *trans-/trans-*, *trans-/cis-*, *cis-/trans-*, *cis-/cis*-rotamer), 66.9, 66.8 (1C, Fmoc-CH_2 *trans-/trans-*, *trans-/cis-*, *cis-/trans-*, *cis-/cis*-rotamer), 62.2, 62.1, 62.0, 61.9, 61.9, 61.7 (2C, C-6, C-6' *trans-/trans-*, *trans-/cis-*, *cis-/trans-*, *cis-/cis*-rotamer), 53.0, 52.7, 52.7, 52.6 (1C, C-2' *trans-/trans-*, *trans-/cis-*, *cis-/trans-*, *cis-/cis*-rotamer), 51.0, 50.8, 50.3, (2C, $NCH_2CO_2{}^tBu$, NCH_2CONH *trans-/trans-*, *trans-/cis-*, *cis-/trans-*, *cis-/cis*-rotamer), 49.0, 48.4 (2C, $COCH_2CO$, $COCH_2$'CO *trans-/trans-*, *trans-/cis-*, *cis-/trans-*, *cis-/cis*-rotamer), 47.3, 46.3 (1C, Fmoc-CH *trans-/trans-*, *trans-/cis-*, *cis-/trans-*, *cis-/cis*-rotamer), 41.7, 41.7, 41.3, 41.2, 39.8, 39.8, 39.7, 39.6, 39.5, 36.7, 36.7, 36.7, (4C, $NHCH_2CH_2N$, $NHCH_2$'CH_2'N *trans-/trans-*, *trans-/cis-*, *cis-/trans-*, *cis-/cis*-rotamer), 28.2, 28.1, 28.1 (12C, $CO_2C(CH_3)_3$ *trans-/trans-*, *trans-/cis-*, *cis-/trans-*, *cis-/cis*-rotamer), 23.2, 22.9, 20.9, 20.9, 20.8, 20.8, 20.7, 20.6 (8C, CH_3 *trans-/trans-*, *trans-/cis-*, *cis-/trans-*, *cis-/cis*-rotamer). Due to the rotameric structure the signals can be exchanged. ESI-TOF-MS: Calcd. for $C_{61}H_{79}N_7O_{26}Na$ $[M+Na]^+$: *m/z* 1348.49670*;* found: *m/z* 1348.49739.

References

1. Thomson, S. A.; Josey, J. A.; Cadilla, R.; Gaul, M. D.; Hassman, F. C.; Luzzio, M. J.; Pipe, A. J.; Reed, K. L.; Ricca, D. J.; Wiethe, R. W.; Noble, S. A.; *Tetrahedron* **1995**, *51(22)*, 6179-6194.
2. Nörrlinger, M.; Ziegler, T.; *Beilstein J. Org. Chem.* **2014**, *10*, 2453-2460.
