# Supplementary material for: Synthesis and NMR studies of malonyl-linked glycoconjugates of N-(2-aminoethyl)glycine. Building blocks for the construction of combinatorial glycopeptide libraries
Source: Beilstein J Org Chem. 2016 Aug 30;12:1939–48. doi: 10.3762/bjoc.12.183 (PMC5082468; doi:10.3762/bjoc.12.183)
Supplement: File 2 — NMR spectra of building blocks 1a–d, 2a–d, 3, 4 and 7; 2D NMR spectra of building block 1a. [file Beilstein_J_Org_Chem-12-1939-s002.pdf]

Supporting Information

for

Synthesis and NMR-studies of malonyl-linked glycoconjugates of *N*-(2-aminoethyl)glycine. Building blocks for the construction of combinatorial glycopeptide libraries

Markus Nörrlinger, Sven Hafner and Thomas Ziegler*

Address: Institute of Organic Chemistry, University of Tuebingen, Auf der Morgenstelle 18, 72076 Tuebingen, Germany

Email: Thomas Ziegler - thomas.ziegler@uni-tuebingen.de
*Corresponding author

NMR spectra of building blocks 1a–d, 2a–d, 3, 4 and 7; 2D NMR spectra of building block 1a

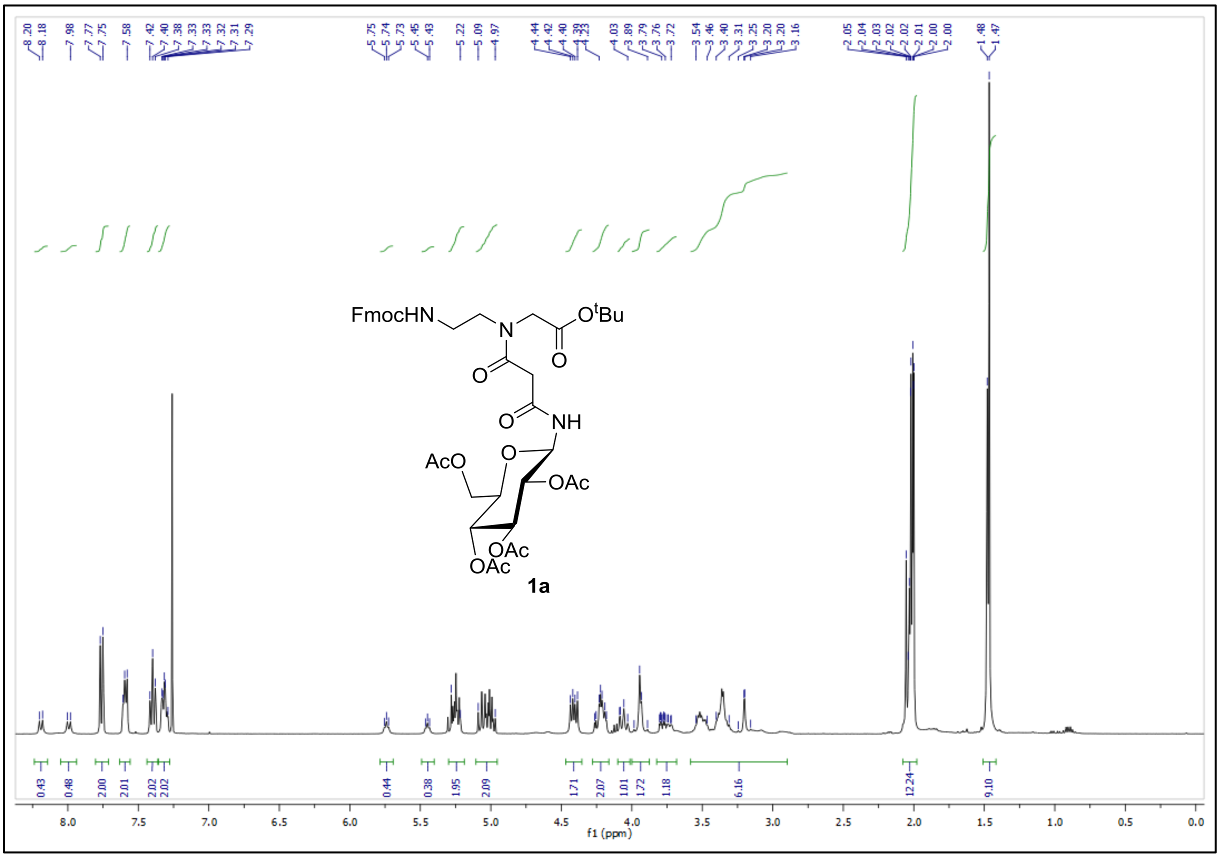
FmocHN
N
O^tBu
O
O
O
NH
AcO
O
OAc
OAc
OAc
1a
f1 (ppm)

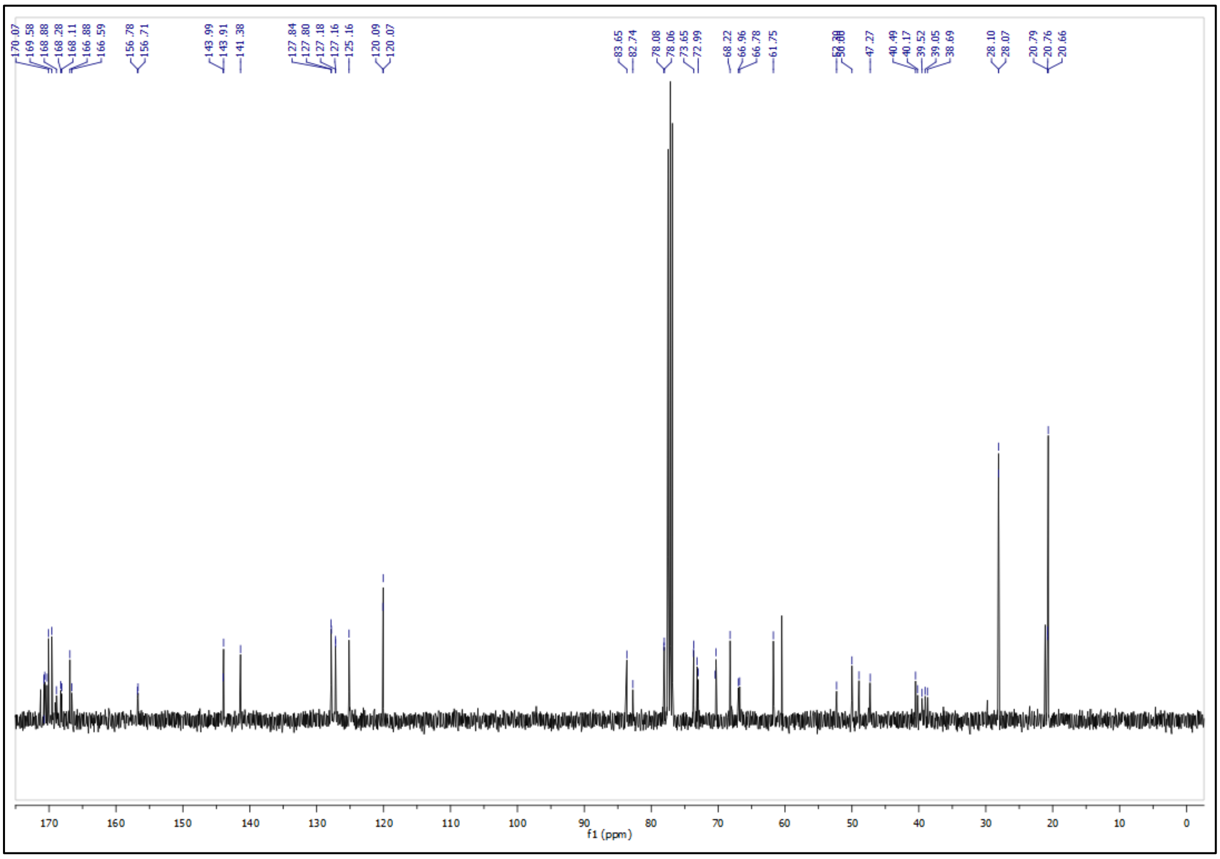
f1 (ppm)

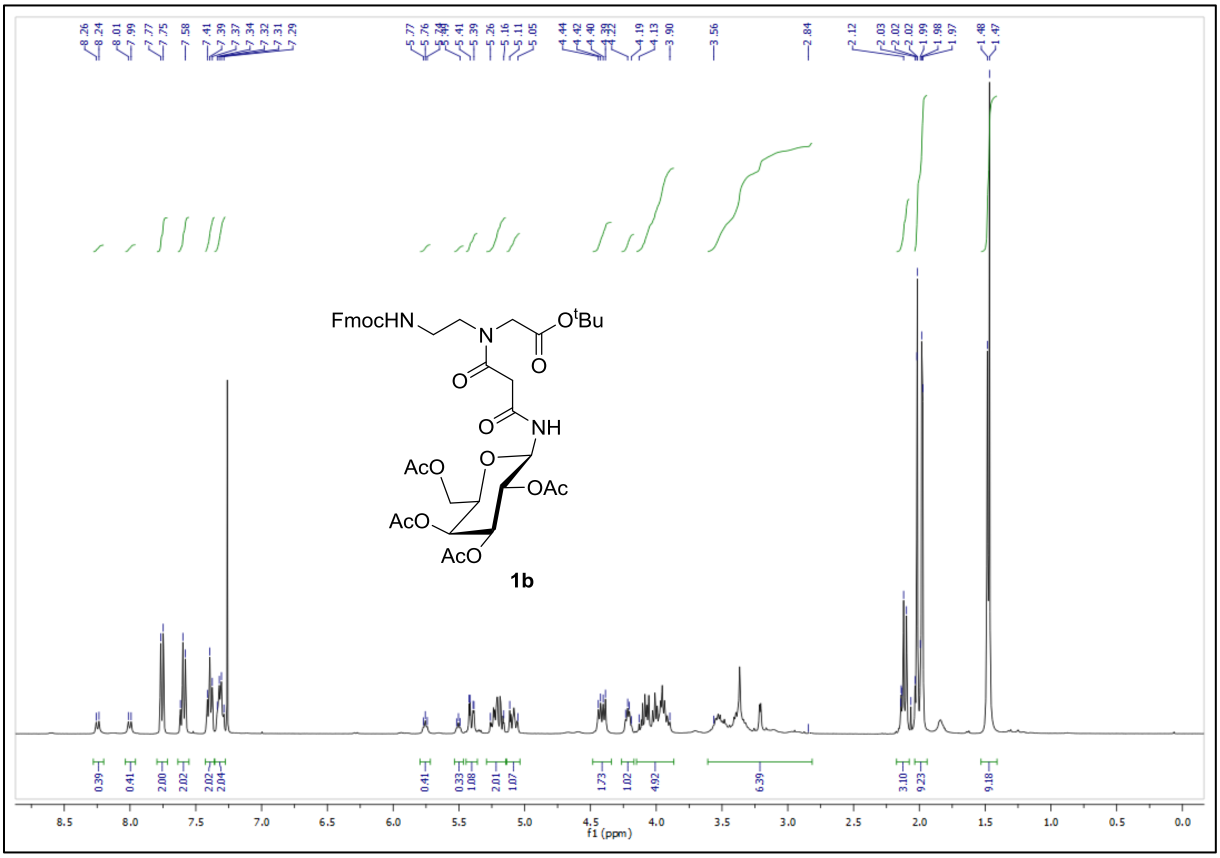

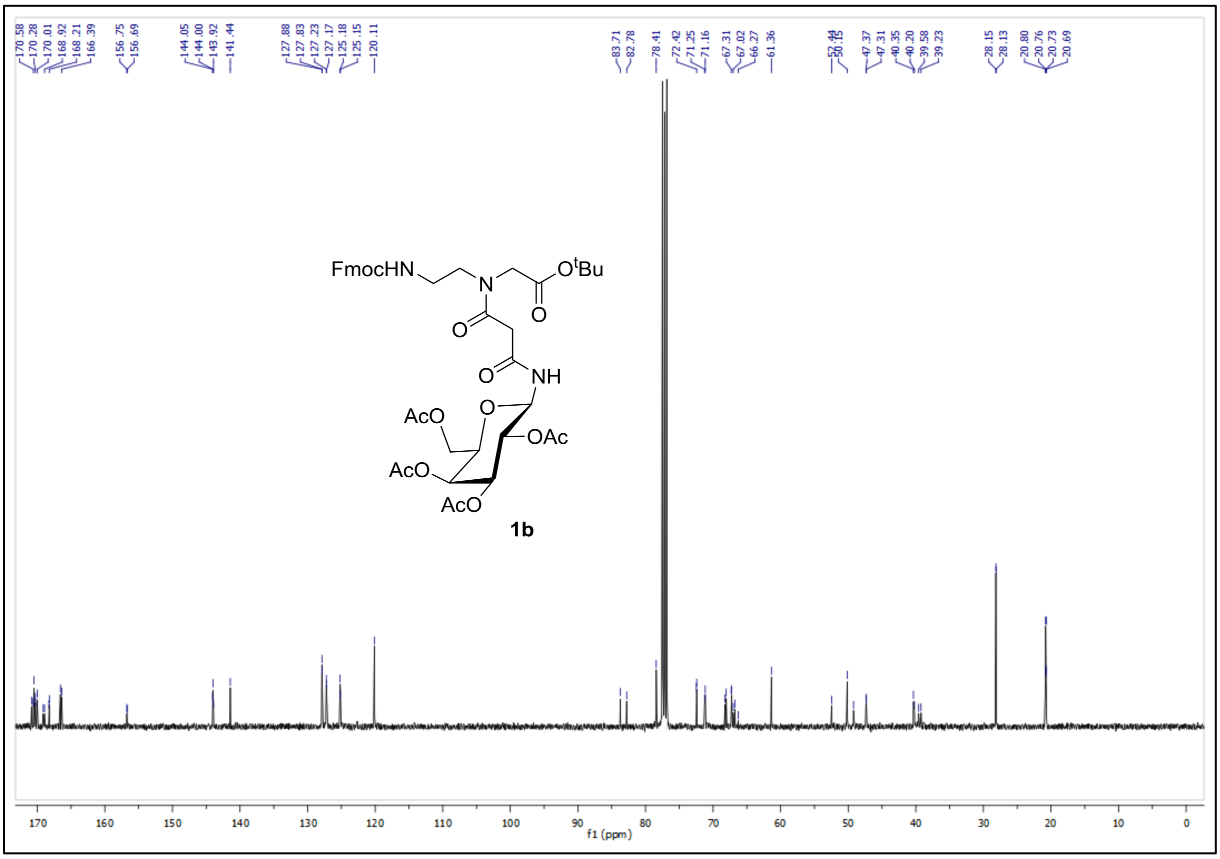

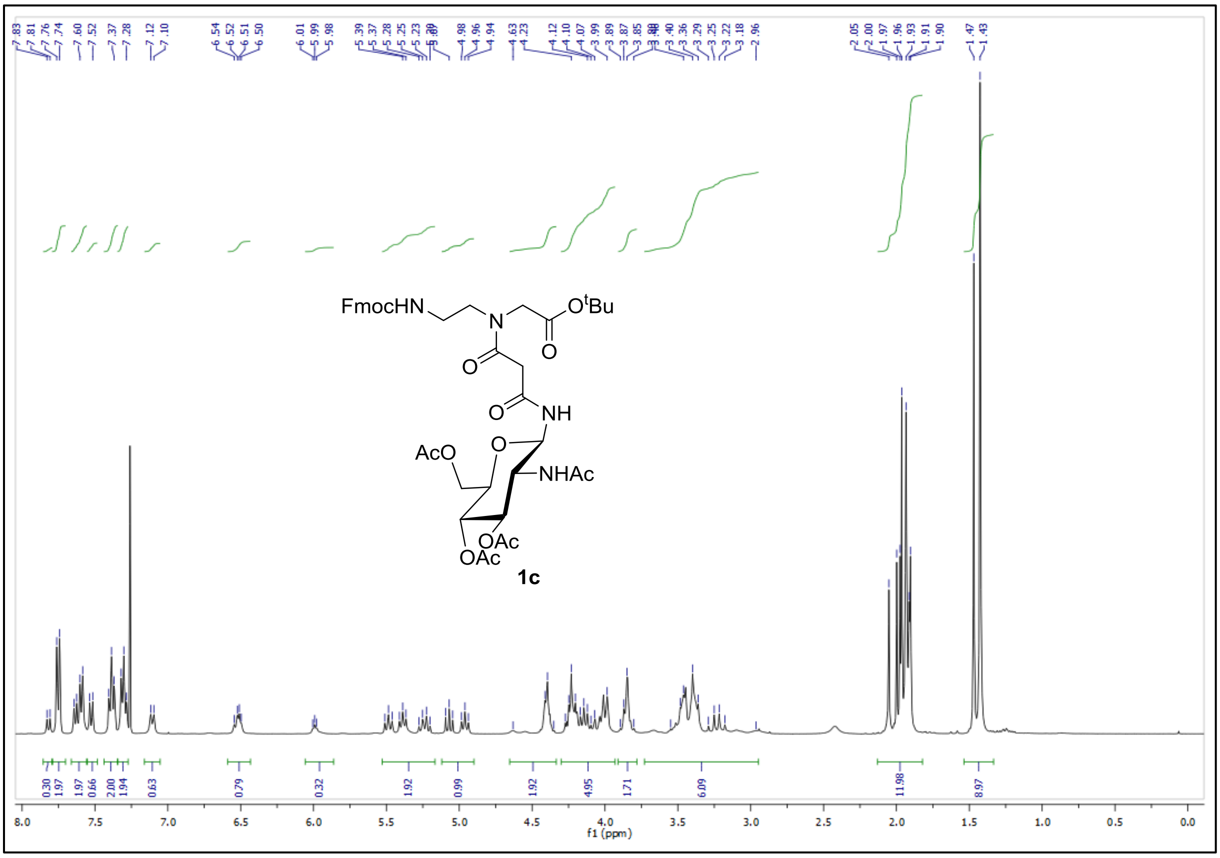

FmocHN
N
O^tBu
O
O
O
NH
AcO
O
NHAc
OAc
OAc
1c
f1 (ppm)

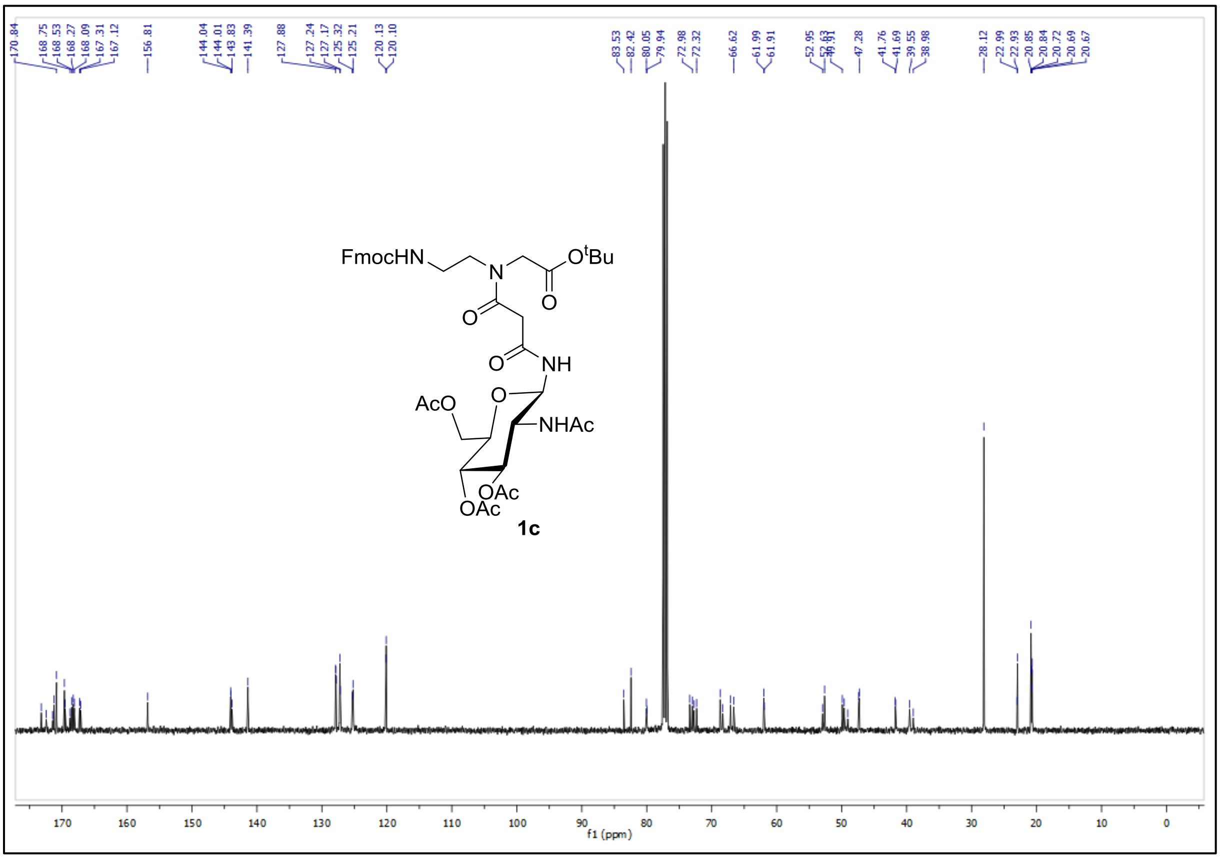

FmocHN
N
O^tBu
O
O
O
NH
AcO
O
NHAc
OAc
OAc
1c
f1 (ppm)

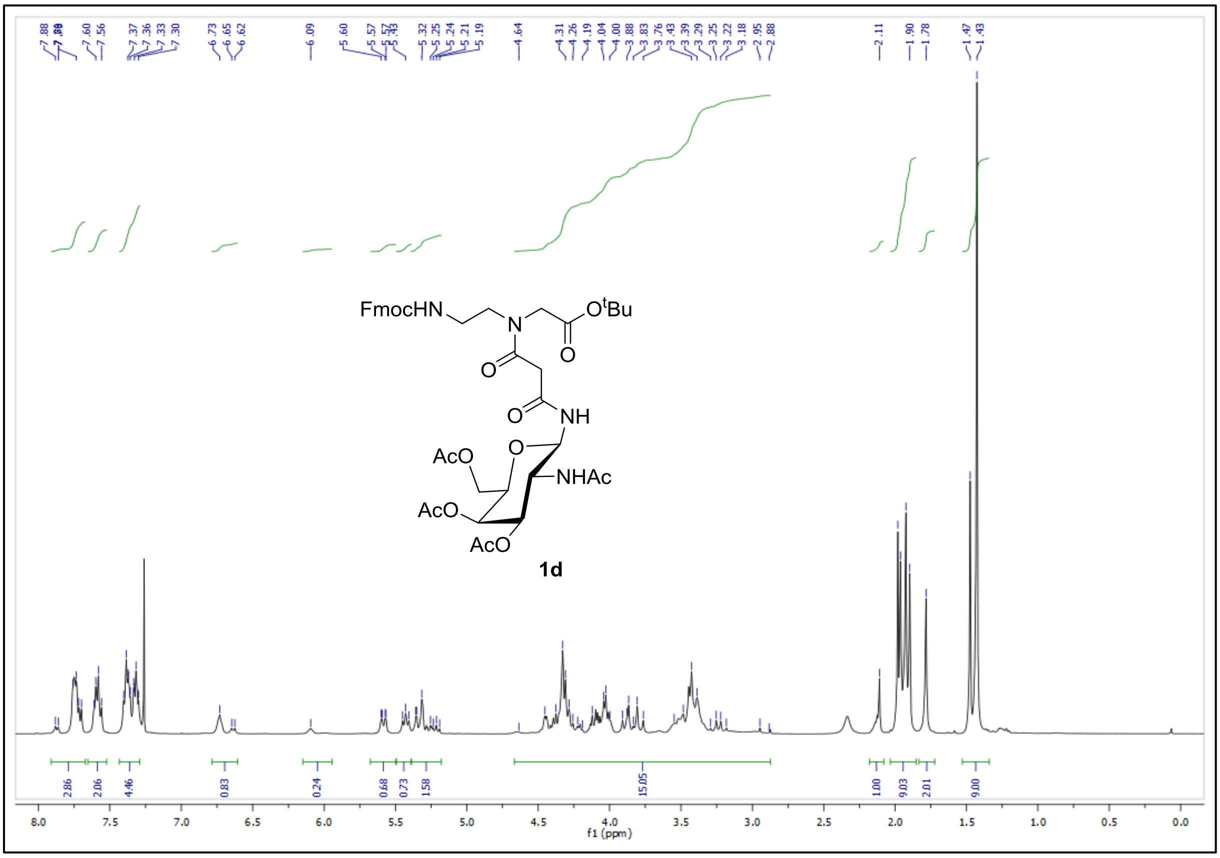

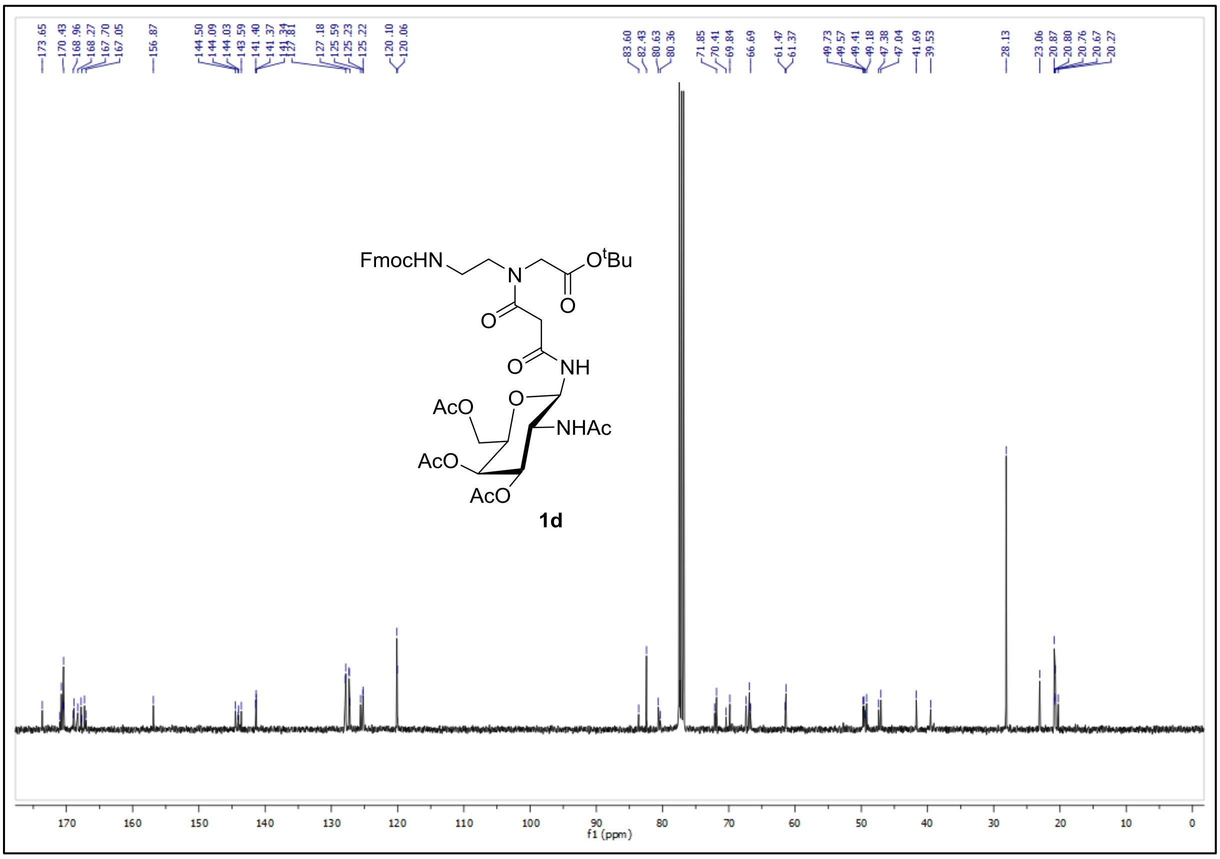

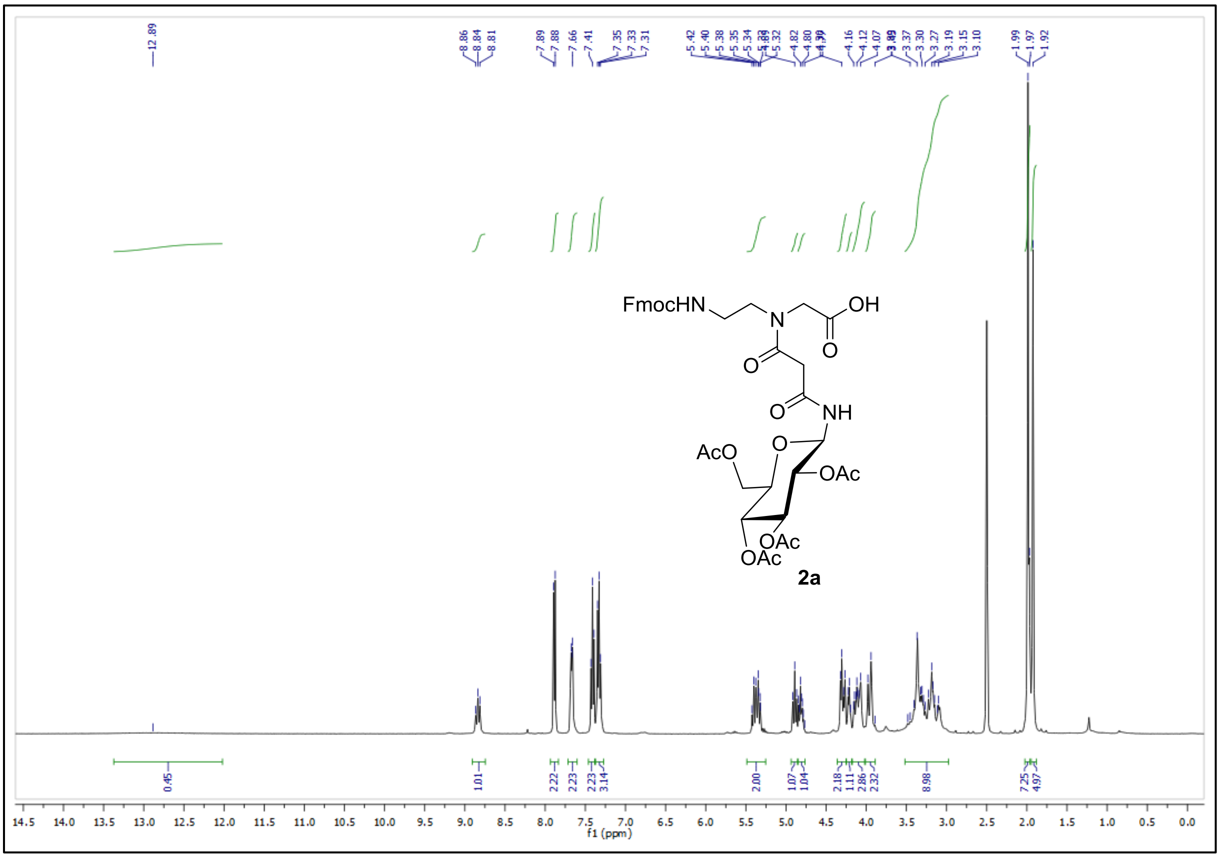
FmocHN
N
OH
O
O
O
NH
AcO
O
OAc
OAc
OAc
2a
f1 (ppm)

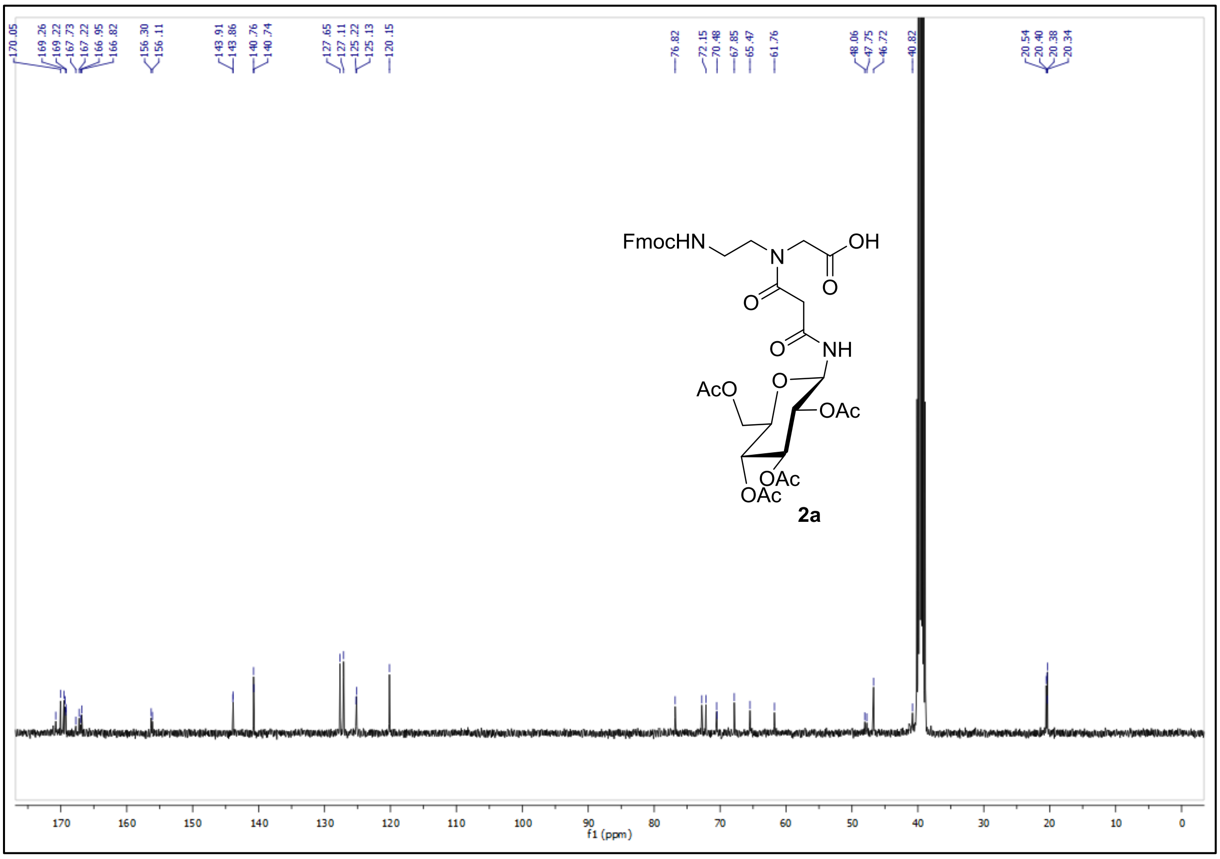
FmocHN
N
OH
O
O
O
NH
AcO
O
OAc
OAc
OAc
2a
f1 (ppm)

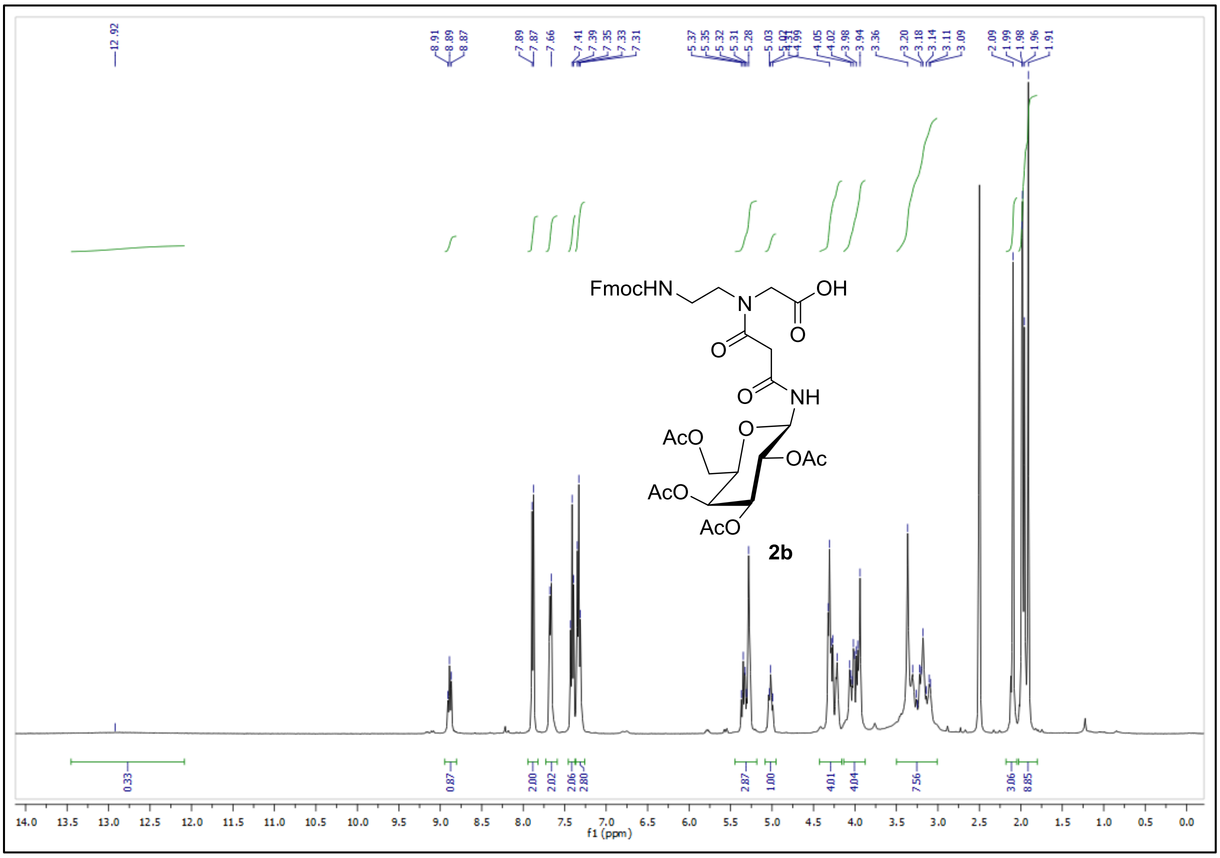

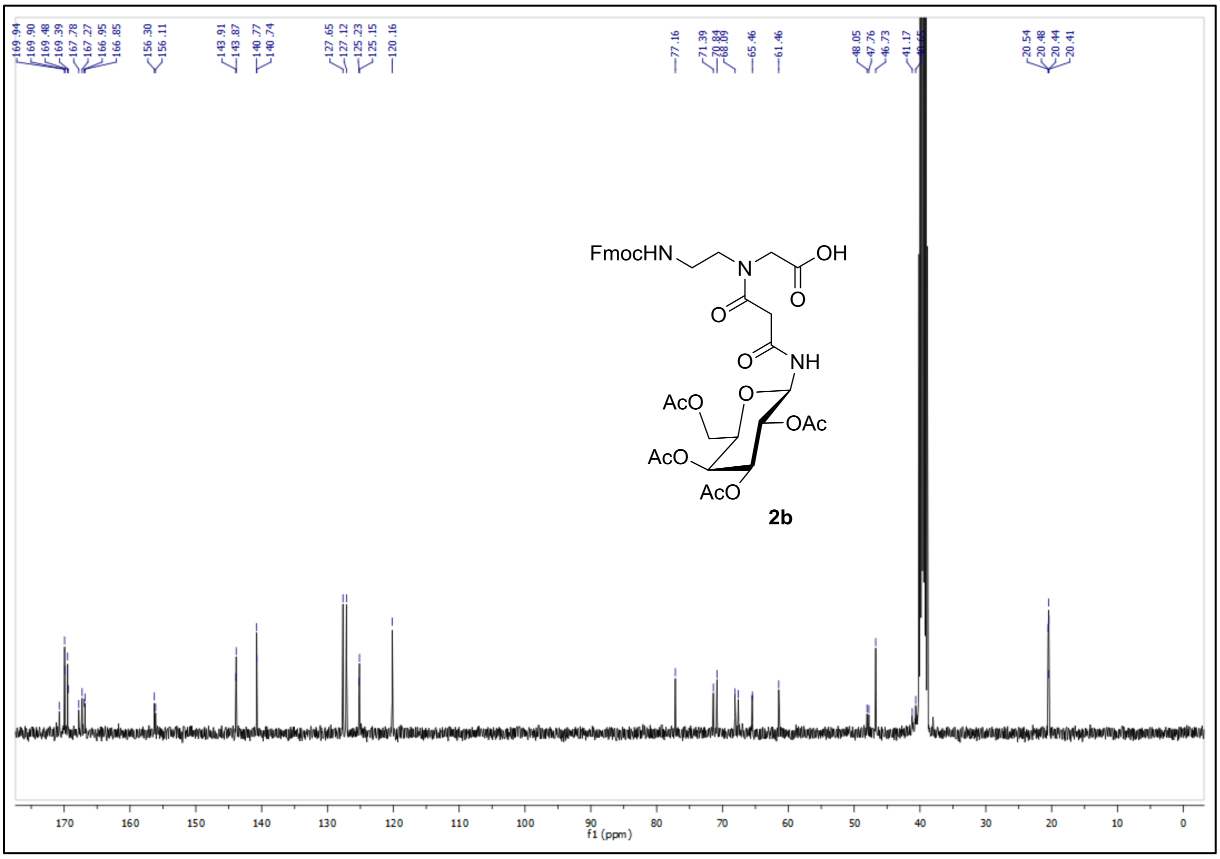

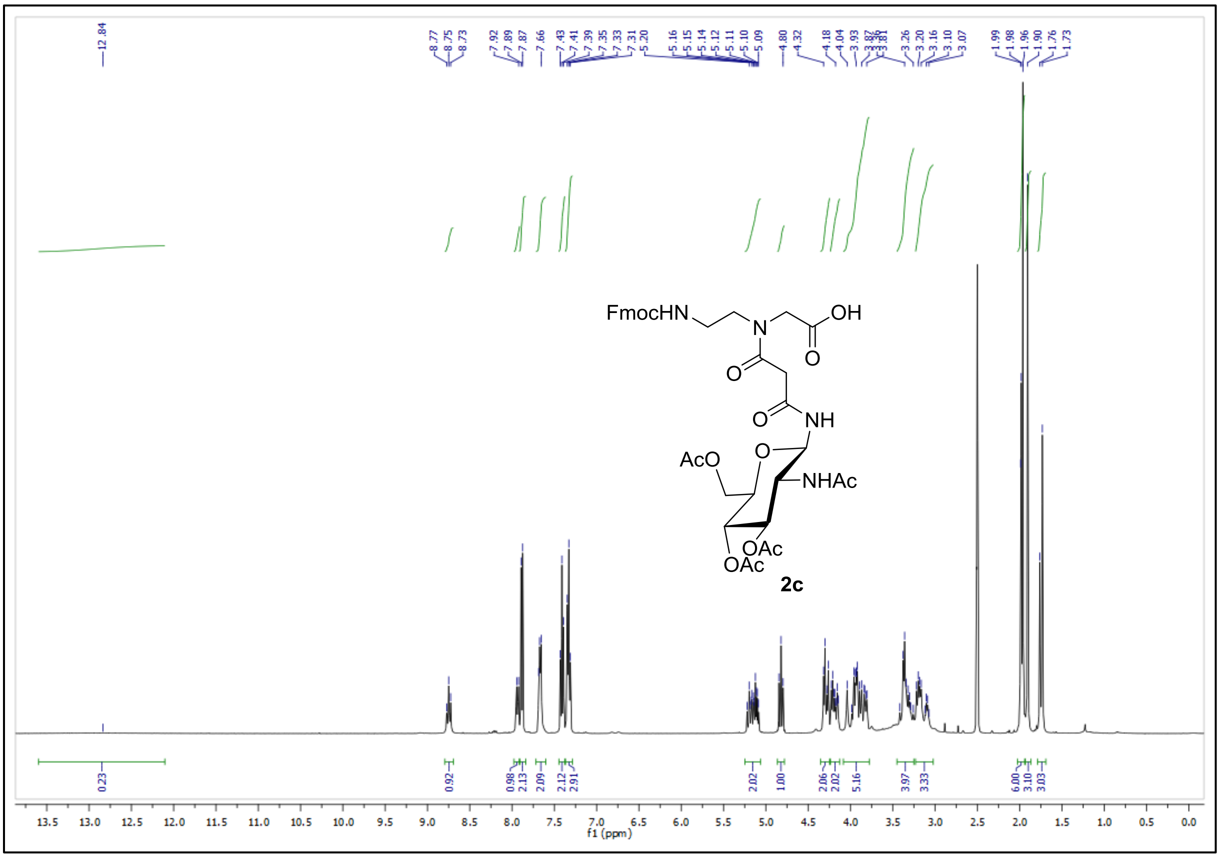

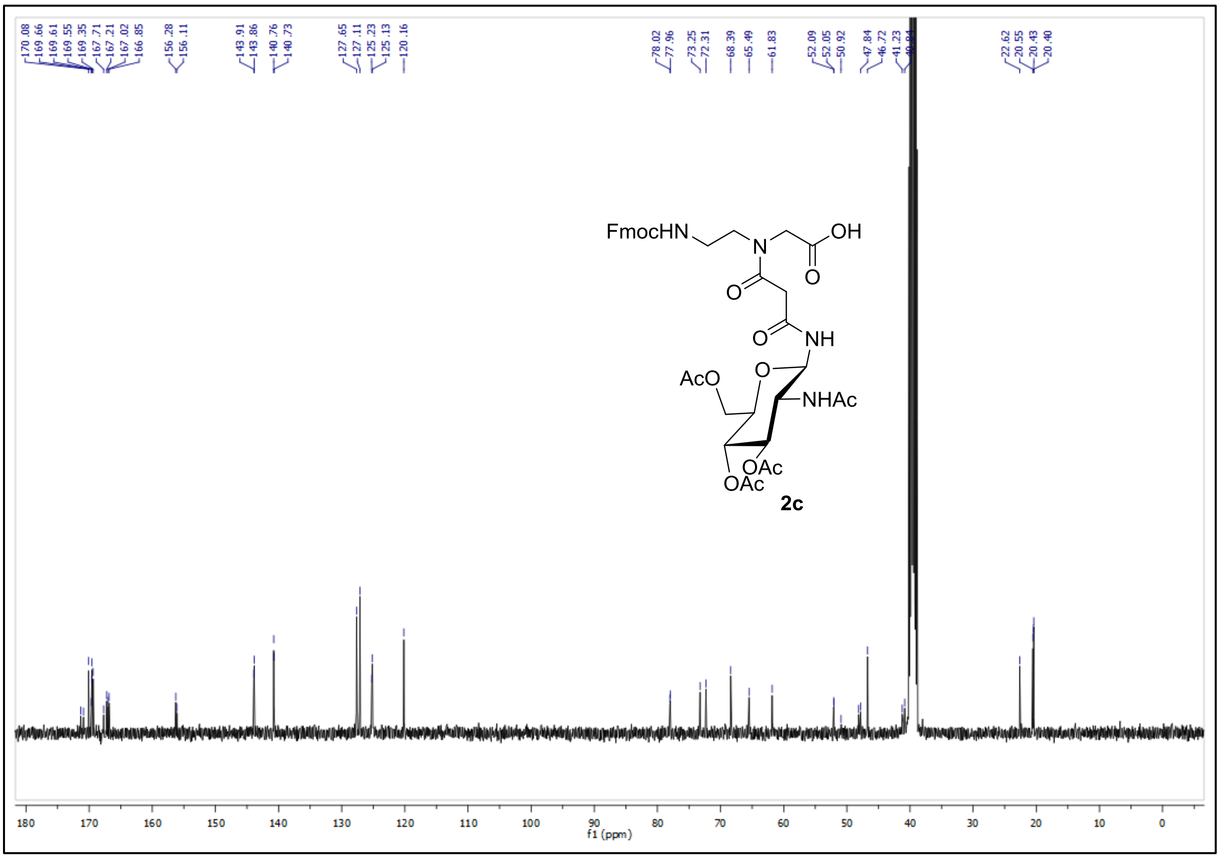

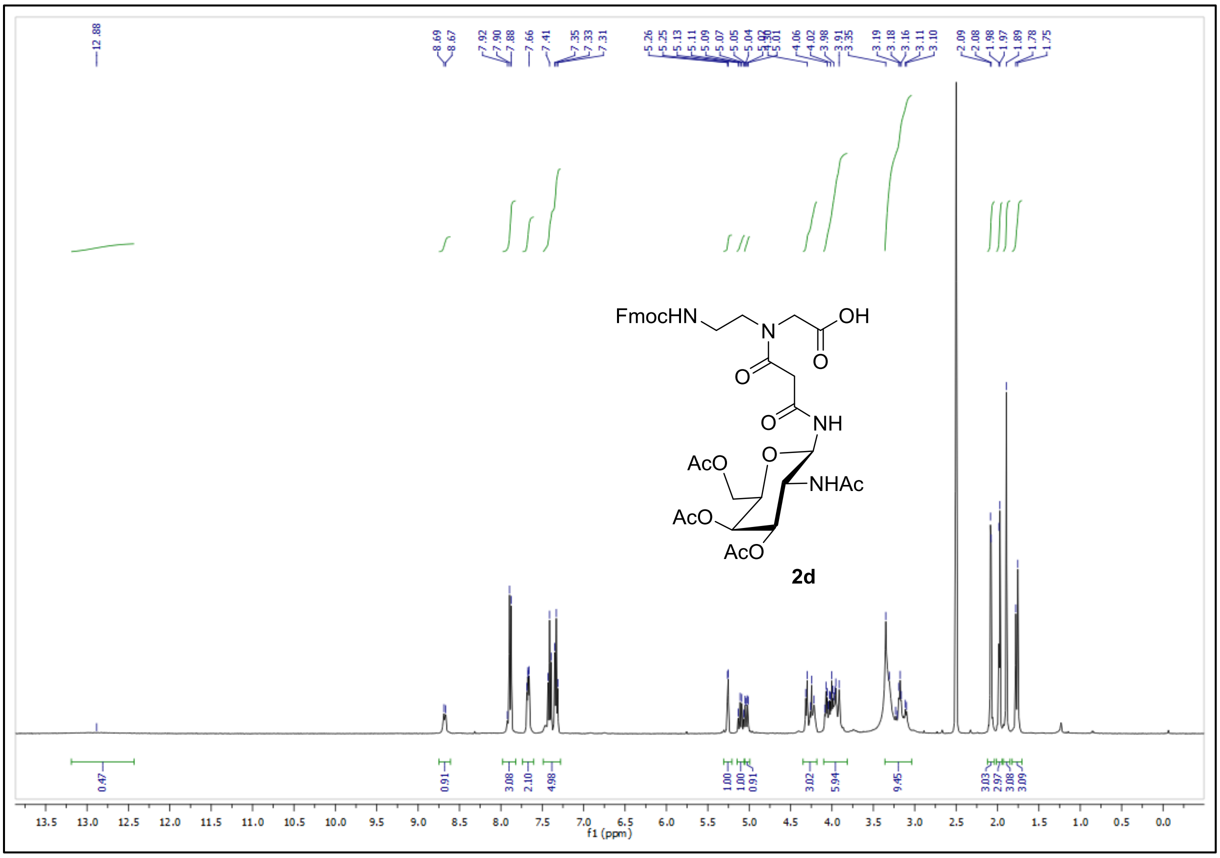
FmocHN
N
OH
O
O
O
NH
AcO
O
NHAc
AcO
AcO
2d
f1 (ppm)

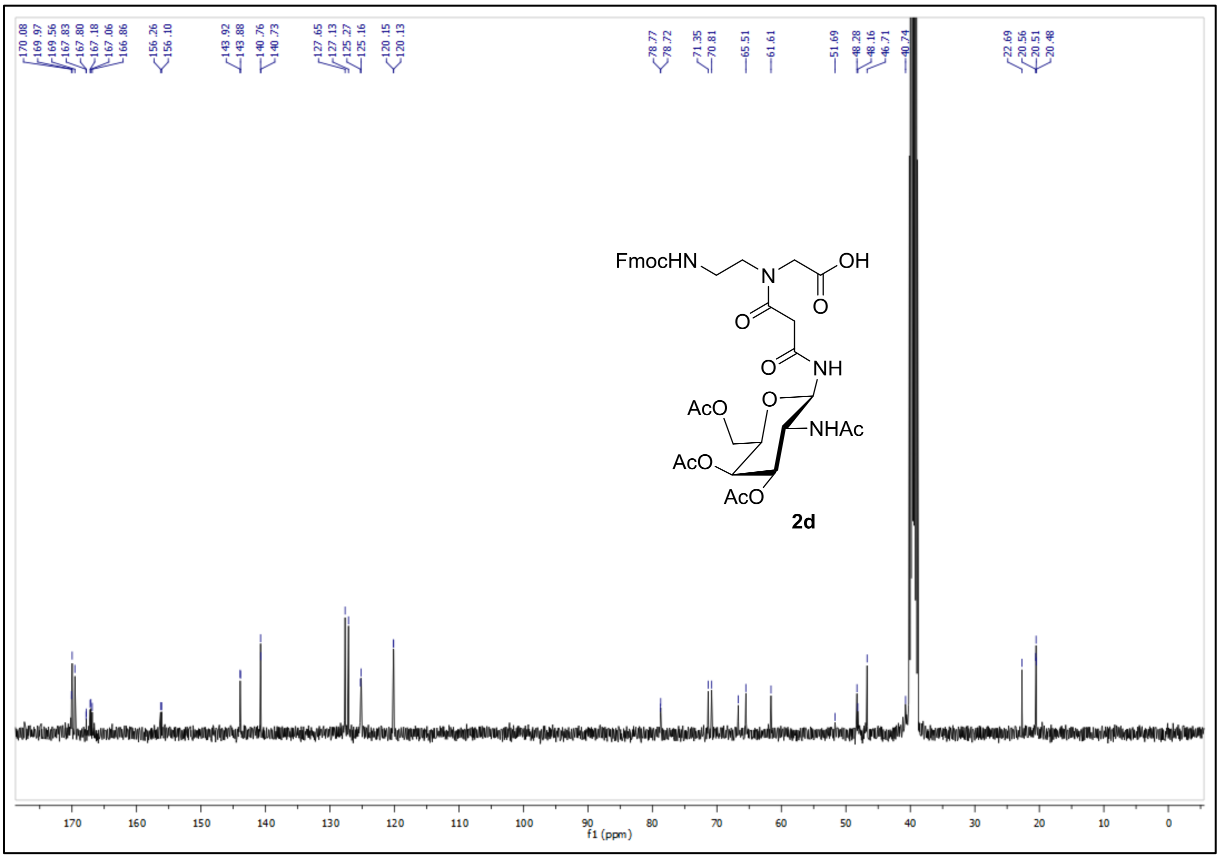
FmocHN
N
OH
O
O
O
NH
AcO
O
NHAc
AcO
AcO
2d
f1 (ppm)

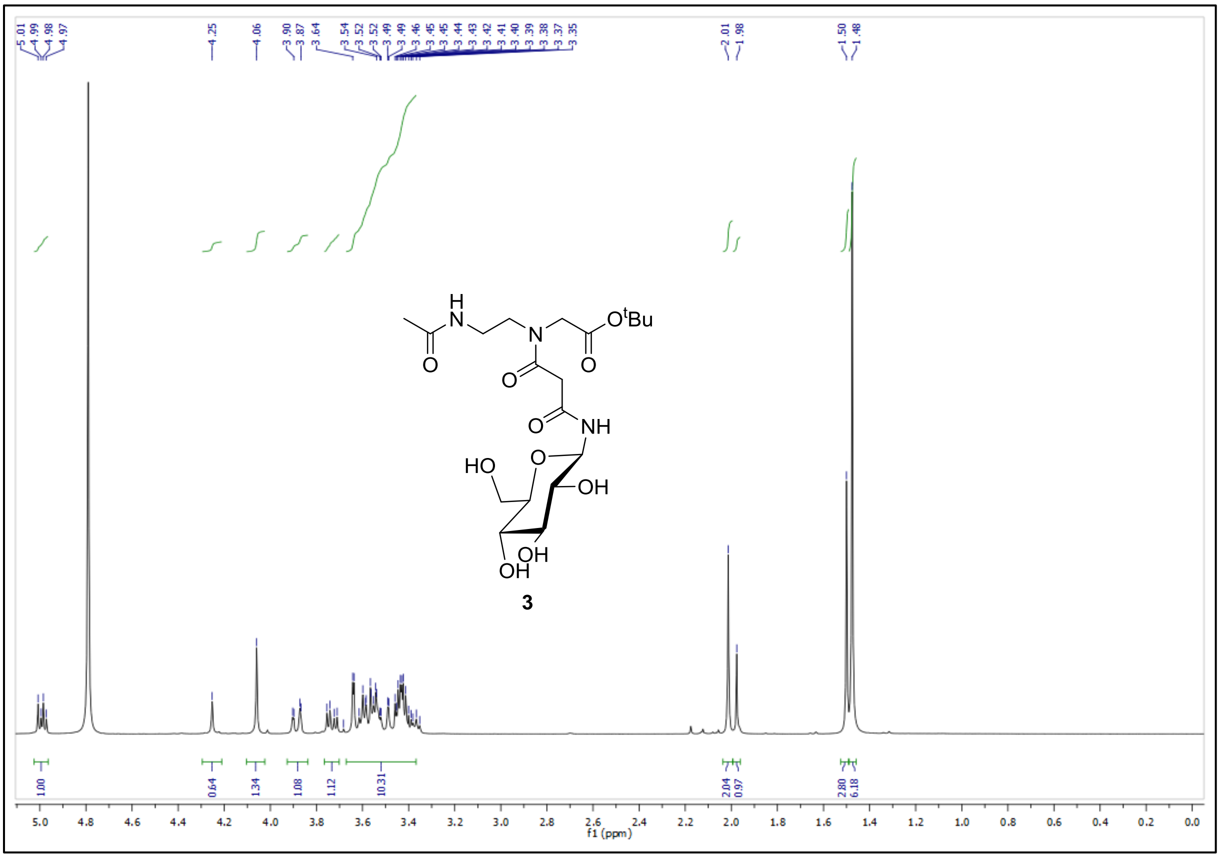
5.01 4.99 4.98 4.97
4.25
4.06
3.90 3.87 3.64 3.54 3.52 3.52 3.49 3.49 3.46 3.45 3.45 3.44 3.43 3.42 3.41 3.40 3.39 3.38 3.37 3.35
2.01 1.98
1.50 1.48
1.00 0.64 1.34 1.08 1.12 10.31 2.04 0.97 2.80 6.18
f1 (ppm)
3

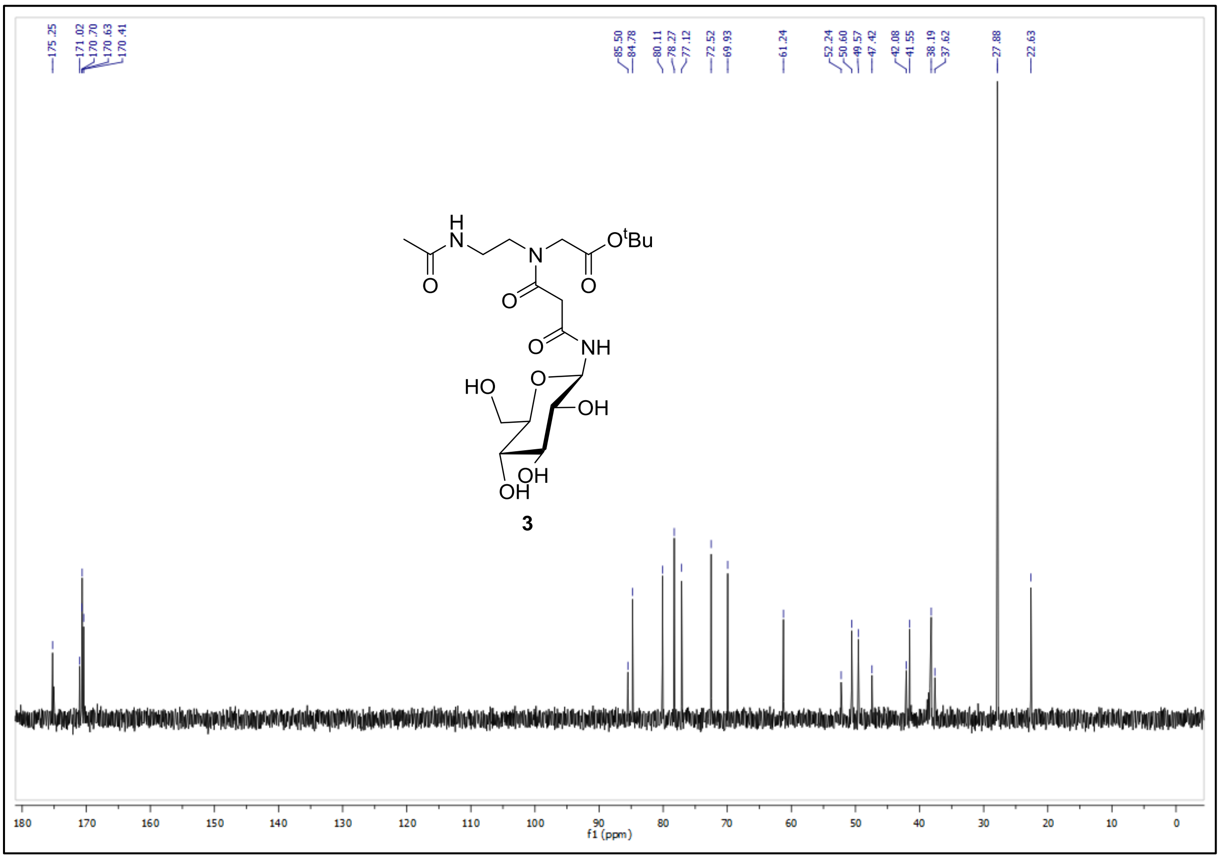
175.25
171.02 170.70 170.63 170.41
85.50 84.78
80.11 78.27 77.12
72.52 69.93
61.24
52.24 50.60 49.57 47.42
42.08 41.55 38.19 37.62
27.88
22.63
f1 (ppm)
3

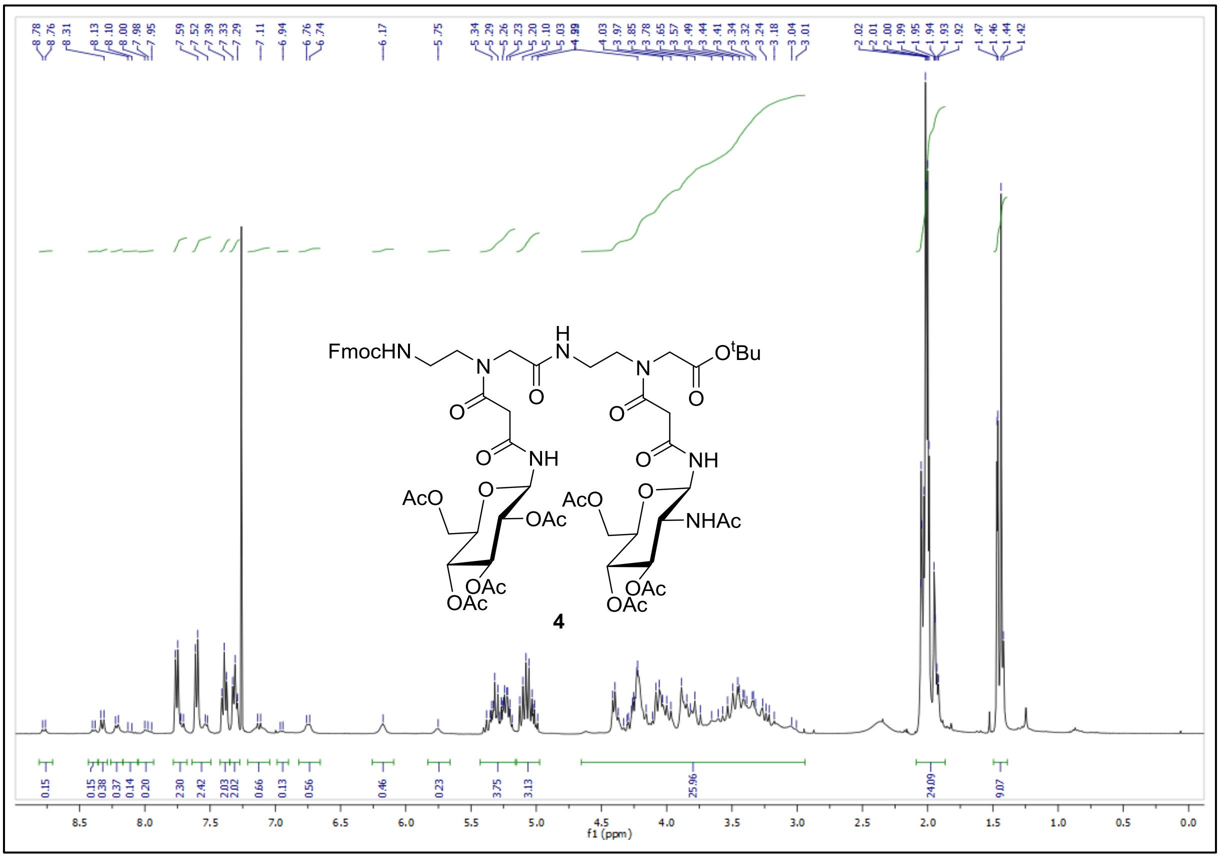

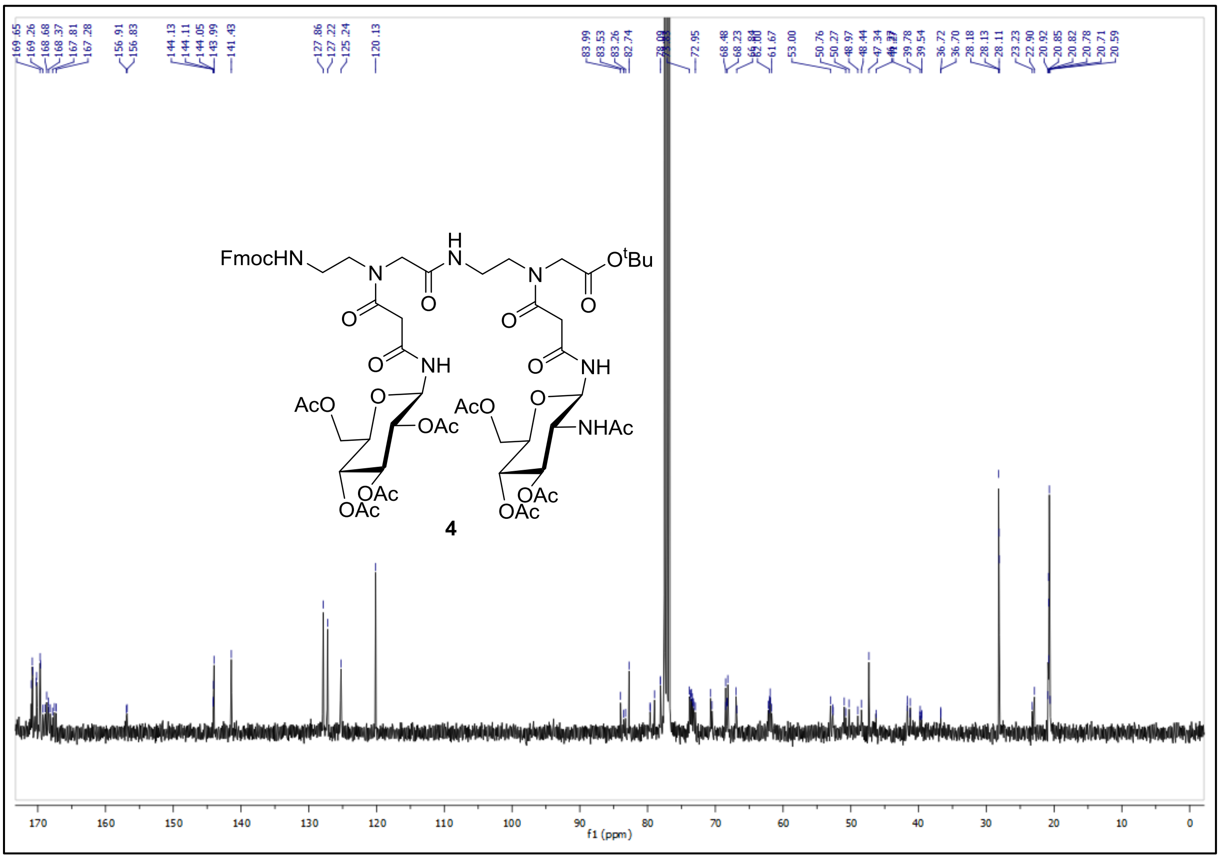

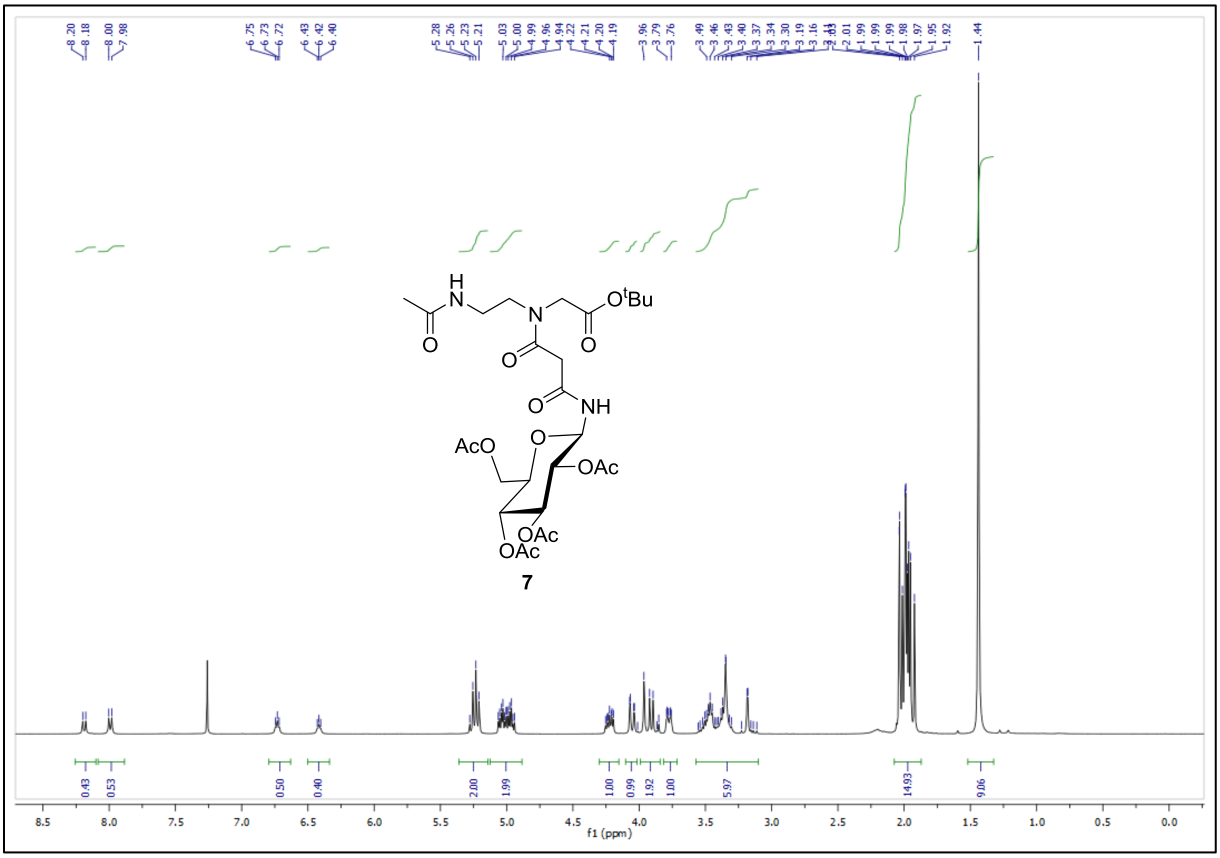

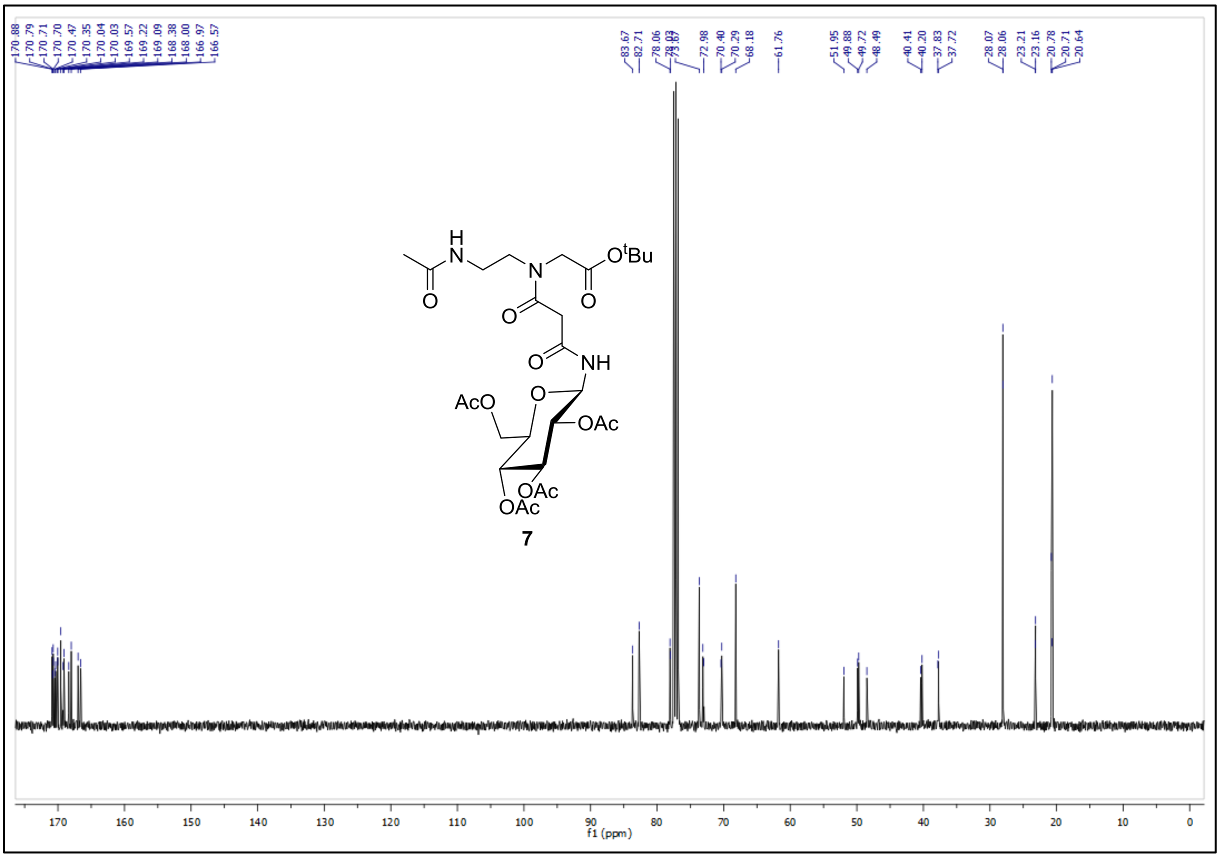

2D NMR Spectra of building block 1a

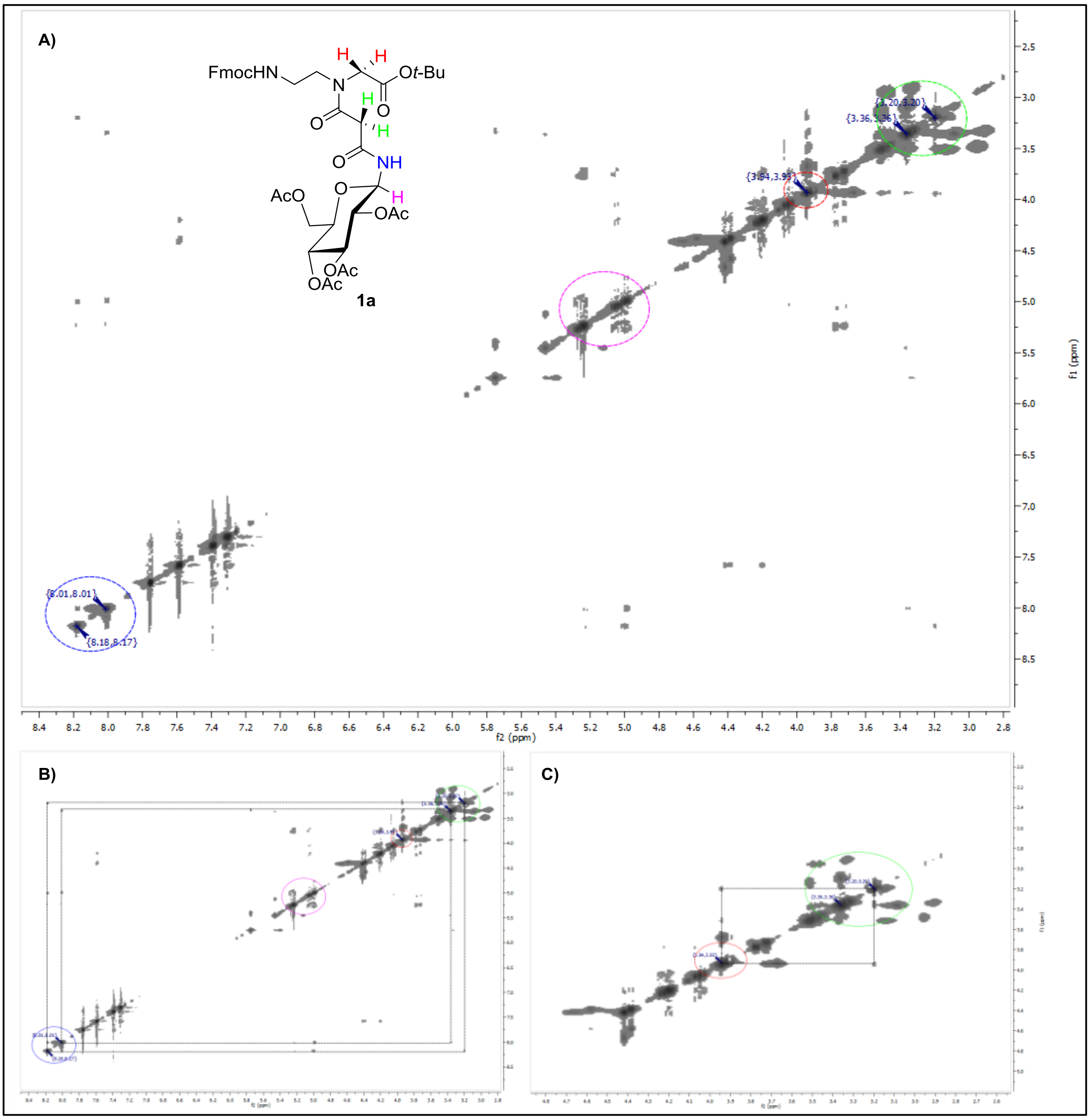

A) 2D-NOESY (600 MHz; τ_m = 450 ms) spectrum of building block **1a** in $CDCl_3$; **B)** Crosspeaks of the amidic doublets (blue) and the malonyl protons (green); **C)** Crosspeaks of the methylene protons of the PNA backbone (red) and the malonyl protons (green).
